# LUX ARRHYTHMO Interacts With ELF3a and ELF4a to Coordinate Vegetative Growth and Photoperiodic Flowering in Rice

**DOI:** 10.3389/fpls.2022.853042

**Published:** 2022-03-17

**Authors:** Zhengzheng Cai, Yudan Zhang, Weiqi Tang, Xuequn Chen, Chenchen Lin, Yang Liu, Yanfang Ye, Weiren Wu, Yuanlin Duan

**Affiliations:** ^1^Key Laboratory of Genetics, Breeding and Multiple Utilization of Crops, Ministry of Education, Fujian Agriculture and Forestry University, Fuzhou, China; ^2^Fujian Key Laboratory of Crop Breeding by Design, Fujian Agriculture and Forestry University, Fuzhou, China

**Keywords:** rice, vegetative growth, photoperiodic flowering, *LUX ARRHYTHMO*, evening complex

## Abstract

The evening complex (EC) plays a critical role in photoperiod flowering in *Arabidopsis*. Nevertheless, the underlying functions of individual components and coordinate regulation mechanism of EC genes in rice flowering remain to be elucidated. Here, we characterized the critical role of *LUX ARRHYTHMO* (*LUX*) in photoperiod perception and coordinating vegetative growth and flowering in rice. Non-functional alleles of *OsLUX* extremely extended vegetative phase, leading to photoperiod-insensitive late flowering and great increase of grain yield. *OsLUX* displayed an obvious diurnal rhythm expression with the peak at dusk and promoted rice flowering *via* coordinating the expression of genes associated with the circadian clock and the output integrators of photoperiodic flowering. OsLUX combined with OsELF4a and OsELF3a or OsELF3b to form two ECs, of which the OsLUX-OsELF3a-OsELF4a was likely the dominant promoter for photoperiodic flowering. In addition, *OsELF4a* was also essential for promoting rice flowering. Unlike *OsLUX*, loss *OsELF4a* displayed a marginal influence under short-day (SD) condition, but markedly delayed flowering time under long-day (LD) condition. These results suggest that rice EC genes share the function of promoting flowering. This is agreement with the orthologs of SD plant, but opposite to the counterparts of LD species. Taken together, rice EC genes display similar but not identical function in photoperiodic flowering, probably through regulating gene expression cooperative and independent. These findings facilitate our understanding of photoperiodic flowering in plants, especially the SD crops.

## Introduction

Heading date is a main agronomic trait for cereal crops to adapt to specific cropping seasons and sophisticate regional environments. Photoperiod pathway is the main regulation pattern for flowering determined by synchronizing the exogenous environmental signals and the endogenous signaling cascades of photoreceptors, circadian clock systems, and floral integrator genes in rice. In *Arabidopsis*, a facultative long-day (LD) plant, the photoperiodic flowering is determined by the *GI* (*GIGANTEA*)-*CO* (*CONSTANS*)-*FT* (*FLOWERING LOCUS T*) pathway. *FT* is a major output of the photoperiodic flowering pathway, and *CO* is a crucial integrator of inducting the photoperiodic expression of *FT*, while *GI* acts both directly and indirectly to stimulate flowering, primarily by promoting *CO* and *FT* expression (Putterill et al., 1995; Park et al., 1999; Suárez-López et al., 2001; Turck et al., 2008; Sawa and Kay, 2011; Shim and Imaizumi, 2015). Although rice normally flowers under short-day (SD) condition, it has also developed the ability of flowering under LD condition. Extensive studies in rice have uncovered the multiple major genetic components and the mechanisms for regulating heading date, including the *OsGI*-*Hd1* (*Heading date 1*)-*Hd3a* (*Heading date 3a*) pathway under SD condition (similar to *Arabidopsis GI*-*CO*-*FT*), and an unique *Ghd7* (*Grain number*, *plant height*, *and heading date 7*)-*Ehd1* (*Early heading date 1*)-*Hd3a*/*RFT1* (*RICE FLOWERING LOCUS T1*) pathway under LD condition (Yano et al., 2000; Kojima et al., 2002; Hayama et al., 2003; Xue et al., 2008; Tsuji et al., 2011; Song et al., 2015; Wei et al., 2020). Unlike *CO*, which promotes flowering in *Arabidopsis*, *Hd1* promotes flowering under SD condition, but represses flowering under LD condition through interaction with *Ghd7* (Yano et al., 2000; Ishikawa et al., 2011; Nemoto et al., 2016).

The circadian clock acts as a hub of photoperiodic flowering in *Arabidopsis*, which directly links flowering to different developmental processes (Bendix et al., 2015). More than 20 clock or clock-associated components have been identified in *Arabidopsis*, which compose multiple interlocking transcription–translation feedback loops (Fogelmark and Troein, 2014; Shim and Imaizumi, 2015). The circadian network is likely conserved in higher plants, but the molecular circuitry that comprises the circadian clock in other plants than *Arabidopsis* has just begun to be understood. In rice, several clock-associated genes have been well characterized, but the circadian clock system still remains largely unclear. *OsGI* contributes to flowering time and the circadian clock and is important for proper regulation of genes within and outside the circadian clock in rice (Izawa, 2012; Shrestha et al., 2014). Knockout mutants of *OsGI* significantly delay flowering under SD condition, but the effect is weak under LD condition (Izawa et al., 2011; Lee and An, 2015), whereas overexpression of *OsGI* in rice causes late flowering regardless of day length, which is accompanied by increased expression of *Hd1* and reduced expression of *Hd3a* (Hayama et al., 2003). Rice *OsPRR37* (*Pseudo-Response Regulator37*) */Hd2/DTH7* downregulates *Hd3a* expression to suppress flowering under LD condition (Koo et al., 2013), and its mutation causes late flowering under SD condition but early flowering under LD condition (Lin et al., 2000). Nonetheless, it is still unclear whether other *OsPRR* genes are required for heading date control. The rice *ELF3* (*EARLY FLOWERING 3*) orthologs, *OsELF3-1/Hd17/Ef7* and *OsELF3-2/OsEF3*, are likely the component of evening complex (EC). Here, we rename *OsELF3-1* and *OsELF3-2* as *OsELF3a* and *OsELF3b*, respectively.

In *Arabidopsis*, EC is a trimeric protein consisting of LUX-ELF3-ELF4 (Nusinow et al., 2011; Silva et al., 2020). LUX is recognized as a transcription factor (Onai and Ishiura, 2005), while ELF3 and ELF4 are unique plant-specific proteins without conserved functional domains (Hicks, 2001; Doyle et al., 2002). EC components showed similar expression profiles, and their mutant alleles share multiple similar phenotypes, including an arrhythmic circadian oscillator, abnormal hypocotyl growth under diurnal cycles, and early flowering (Nusinow et al., 2011). EC is a direct regulator of genes. It represses the expression of circadian genes *GI*, *PRR7* and *PRR9* in the late evening by binding to the conserved LUX binding site in their promoters (Dixon et al., 2011; Helfer et al., 2011; Chow et al., 2012; Herrero et al., 2012; Mizuno et al., 2014). Meanwhile, EC also represses itself near dawn through inhibition of LUX, which allows the clock regulatory cycle to repeat the next day. This self-negative-feedback regulation of LUX forms an additional loop of EC (Helfer et al., 2011; Chow et al., 2012; Mizuno et al., 2014).

All of the EC components can be found in the genomes of land plant lineages. So far, most of our understanding of EC function comes from *Arabidopsis*, while the information about EC orthologs remains limited in other plants (Nusinow et al., 2011; Huang and Nusinow, 2016). However, it is likely that the ECs in other species than *Arabidopsis* will interact with different proteins/pathways or regulate new outputs. Therefore, molecular understanding of how EC gene orthologs function in other plants, especially in SD crops, such as rice, corn, and sorghum, will be necessary for elucidating their roles in circadian- and photo-regulation of physiology (Huang and Nusinow, 2016). Recently, two putative evening complexes have been identified and the accurate function of partial components has been characterized in soybean (Liew et al., 2017; Bu et al., 2021; Fang et al., 2021). In rice, two *ELF3* orthologs have been characterized. Loss of *OsELF3a* function disrupts photoperiod-dependent control of key flowering time genes, and produces a photoperiod-insensitive flowering type (Matsubara et al., 2012; Saito et al., 2012; Zhao et al., 2012; Yang et al., 2013), whereas the contribution of *OsELF3b* to either flowering time or the circadian clock remains controversial (Fu et al., 2009; Zhao et al., 2012).

In this study, we identified *OsLUX* in rice through map-based cloning, characterized the role and interactions of OsLUX in the rice flowering pathway, and identified two rice ternary repressive protein complexes composed of OsLUX OsELF4a, and OsELF3a or OsELF3b. We found that the complex OsLUX-OsELF3a-OsELF4a is likely the dominant promoter for photoperiodic flowering. In addition, we characterized the role of *OsELF4a* in the control of rice heading date using CRISPR/Cas9. Thus, rice is another species apart from *Arabidopsis* and soybean with the components of EC identified and well characterized. The findings of this study provide new insights into the EC function and may facilitate our understanding of the mechanisms of photoperiodic flowering in other SD species.

## Materials and Methods

### Plant Materials

Rice cultivars Nipponbare (*japonica*), DZ60 (*tropical japonica*), *long vegetative phase2-1* (*lvp2-1*) mutant and eight editing lines including *lvp2-2* to *lvp2-7*, *Oself4a-1*, and *Oself4a-2* were used in the study. The *lvp2-1* mutant was obtained from the progeny of EMS mutagenesis of Nipponbare; *lvp2-2* to *lvp2-7*, *Oself4a-1*, and *Oself4a-2* were generated from Nipponbare using the CRISPR/Cas9 system.

### Identification of *LVP2*/*OsLUX* Gene

To map *LVP2* locus, an F_2_ population was developed from the cross between *lvp2-1* and DZ60. To avoid the interfering effects of other flowering genes, a total of 389 progeny lines (F_2:3_) which display extremely late flowering and other phenotypic characteristics similar to *lvp2-1* were selected for gene mapping. *LVP2* was mapped to a rice chromosome by using the publicly available RM-series rice microsatellite markers (McCouch et al., 2002) and bulked segregant analysis (BSA) method. Furthermore, mapping of *LVP2* gene was also performed by deep sequencing-based bulked segregant analysis (BSA-seq) based on an F_3_ population developed from the cross between *lvp2-1* and Nipponbare. To make the wild-type DNA pool and the mutant DNA pool, equal amount of fresh leaves from 30 homozygous wild-type lines (5 plants/line) and 30 mutant lines randomly selected from the F_3_ population were mixed for genomic DNA extraction, respectively. The two DNA pools were sequenced using the Illumina PE Genome Analyzer. Based on the sequence information (read length = 300 bp, sequencing depth > 30×) of the two pools, the *LVP2* locus was mapped.

### Vector Construction and Plant Transformation

For genetic complementation test, a 3,420-bp genomic DNA sequence covering the promoter region, gene region, and downstream region of *OsLUX* was amplified from Nipponbare, and inserted into the binary vector pCAMBIA1300. The plasmid was introduced into *lvp2-1* mutant embryonic calli. To confirm the function of *OsLUX* and to identify the function of *OsELF4a*, the CRISPR/Cas9 system was used, and the CRISPR targets for *OsLUX* and *OsELF4a* were selected as described by Miao et al. (2013). The vector construction was performed according to the manufacturer’s instruction of regent kit (VIEWSOLID Biotech, China). The vectors were then transferred into Nipponbare. The genomic regions surrounding the CRISPR target sites for *OsLUX* and *OsELF4a* were amplified by PCR using primer pairs 5′-tcgagtccccgatttggttc/5′-gcttcacgtagaggcgatact and 5′-atggaaggtgatagcttct/5′-gccgggccggacacgcttc, respectively, and were sequenced to identify mutants. For overexpression test, a 717-bp cDNA of *OsLUX* was amplified and fused into the downstream of maize Ubiquitin promoter of the binary vector *pTCK303*. The plasmid for overexpression was introduced into Nipponbare. To determine the expression pattern of *OsLUX*, a 2021-bp promoter upstream of the coding region of *OsLUX* was amplified from Nipponbare, and fused into the GUS reporter gene with the nopaline synthase terminator in *pCAMBIA1391Z*. The plasmid was introduced into Nipponbare. Histochemical assay for GUS activity in transgenic plants was performed as described (Jefferson et al., 1987).

All constructs were confirmed by sequencing, introduced into *Agrobacterium tumefaciens* strain EHA105, and transferred into the rice variety by *Agrobacterium*-mediated transformation. The primers used for vector construction are listed in Supplementary Table S1.

### Subcellular Localization

OsLUX-GFP fusion was made by in-frame fusion of the 717-bp full-length coding sequence of OsLUX into vector pRTVcGFP (He et al., 2018). OsLUX-GFP and GFP alone (as control) were transferred into rice protoplasts. NLS-RFP was co-transfected for nuclear localization control. The samples were observed with a confocal laser scanning microscope (LEICA-SP8). Primer pairs for amplifying *OsLUX* cDNA are listed in Supplementary Table S1.

### Yeast Two-Hybrid (Y2H)

For Y2H assay, the coding sequences of *OsLUX* and *OsELF4a* were amplified from Nipponbare and inserted into pGBKT7. The coding region of OsELF3a and OsELF3b were amplified from Nipponbare and inserted into pGADT7. Different combinations of bait and prey were used to co-transform the yeast competent cell Y187. The transformed yeast cells were grown on non-selective SD media (−Trp-Leu/−LT) and selective SD media (−Trp-Leu-His-Ade/-LTHA) with X-α-Gal. A positive interaction was judged by the growth of yeast colonies on selective media. The primers used for vector construction are listed in Supplementary Table S1.

### Bimolecular Fluorescence Complementation Assay

The CDSs of *OsLUX*, *OsELF3a*, *OsELF3b*, *OsELF4a*, *OsELF4b*, and *OsELF4c* (without termination codon) were amplified and inserted into the entry vector pDONR207 *via* a BP reaction, and then transferred into the N-terminal and the C-terminal of pSPYNE or pSPYCE (Choi et al., 2012) *via* a LR reaction, to form fusion protein NE-LUX, CE-LUX, NE-ELF3a, NE-ELF3b, CE-ELF4a, CE-ELF4b, and CE-ELF4c, respectively. Equal concentration mixture of NE-ELF3a and CE-LUX, NE-ELF3b and CE-LUX, NE-ELF3a and CE-ELF4a, NE-ELF3b and CE-ELF4a, NE-ELF3a and CE-ELF4b, NE-ELF3b and CE-ELF4b, NE-ELF3a and CE-ELF4c, NE-ELF3b and CE-ELF4c, and NE-LUX and CE-ELF4a were used to co-transform rice protoplasts. The vector combinations of CE + NE-LUX, NE + CE-LUX, CE + NE-ELF3a, CE + NE-ELF3b, NE + CE-ELF4a, NE + CE-ELF4b, and NE + CE-ELF4c were used as negative controls to verify the specificity of the interactions. Fluorescence in transformed protoplast was examined and imaged using a confocal laser scanning microscope (LEICA-SP8). The primers used for vector construction are listed in Supplementary Table S1.

### Co-immunoprecipitation (Co-IP) Assays

To further validate the interaction between OsLUX and interacted partners *in vivo*, Co-IP assay was conducted. The ORFs of *OsLUX* and *OsELF4a* were amplified and inserted into pRTVcGFP (He et al., 2018) to generate expression vectors *pRTVcGFP-OsLUX* and pRTVcGFP-*OsELF4a*. Full-length cDNAs of ELF3a and ELF3b were amplified and cloned into the psuper1300-MYC. The Co-IP assay was performed as described (Kong et al., 2019). Briefly, the plasmids were used to transiently co-transform rice protoplasts from ten-day-old etiolated seedlings. After incubation for 16 h, total proteins were extracted from the protoplasts. Lysis buffer (50 mM Tris-MES pH = 0.8, 0.5Msucrose, 1 mM MgCl2, 10 mM EDTA, 1 mM PMSF, 0.5 mM DTT, 0.1% Trixon-100) was added to protease inhibitor cocktail (Sigma-Aldrich, P9599). The supernatant was collected after centrifugation 16,000 g for 30 min at 4°C and precleared with GFP-Nanoab-Agarose (Lablead, GNA-20-400) for 1 h. The GFP-Nanoab-Agarose was washed four times in 0.5 ml lysis buffer and then boiled in the 2XSDS-PAGE sample buffer. The immunoprecipitation products were detected by SDS-PAGE and western blot using anti-Myc (MBL, M047-3) or anti-GFP (MBL, 598) antibody. The primers used for vector construction are listed in Supplementary Table S1.

### RNA Isolation and qRT-PCR Analysis

RNA isolation and qRT-PCR analysis were performed as described (Duan et al., 2019). Briefly, total RNA was isolated using TRIzol reagent kit (Invitrogen, Carlsbad, CA, United States). Reverse transcription of total RNA was performed using Primescript^™^ RT reagent kit (Takara, China). The cDNA samples were diluted to 8 ng/μl and 2 ng/μl. Triplicate quantitative assays were performed using the SYBR *Premix Ex Taq* II (Takara, China) with a Mastercycler ep realplex^4^ sequence detection system (Eppendorf). Amplification of UBQ was used as internal control to normalize all data. Primers used for qRT-PCR analysis are listed in Supplementary Table S2.

### Accession Numbers

Sequence data from this article for the mRNA, cDNA, and genomic DNA can be found in the GenBank/EMBL/Gramene data libraries or Web site under accession numbers:

*OsLUX*, LOC_Os01g74020; *OsGI*, LOC_Os01g08700; *OsLHY*, LOC_Os08g06110; *OsPRR37*, LOC_Os07g49460; *OsPRR95*, LOC_Os09g36220; *OsELF3a*, LOC_Os06g05060; *OsELF3b*, LOC_Os01g38530; *OsELF4a*, LOC_Os11g40610; *OsELF4b*, LOC_Os03g29680; *OsELF4c*, LOC_Os08g27860; *Hd1*, LOC_Os06g16370; *Hd3a*, LOC_Os06g06320; *Ghd7*, LOC_Os07g15770; *Ehd1*, LOC_Os10g32600.

## Results

### *lvp2-1* Exhibited Extremely Late Flowering Regardless of Day Length

**Figure 1:**
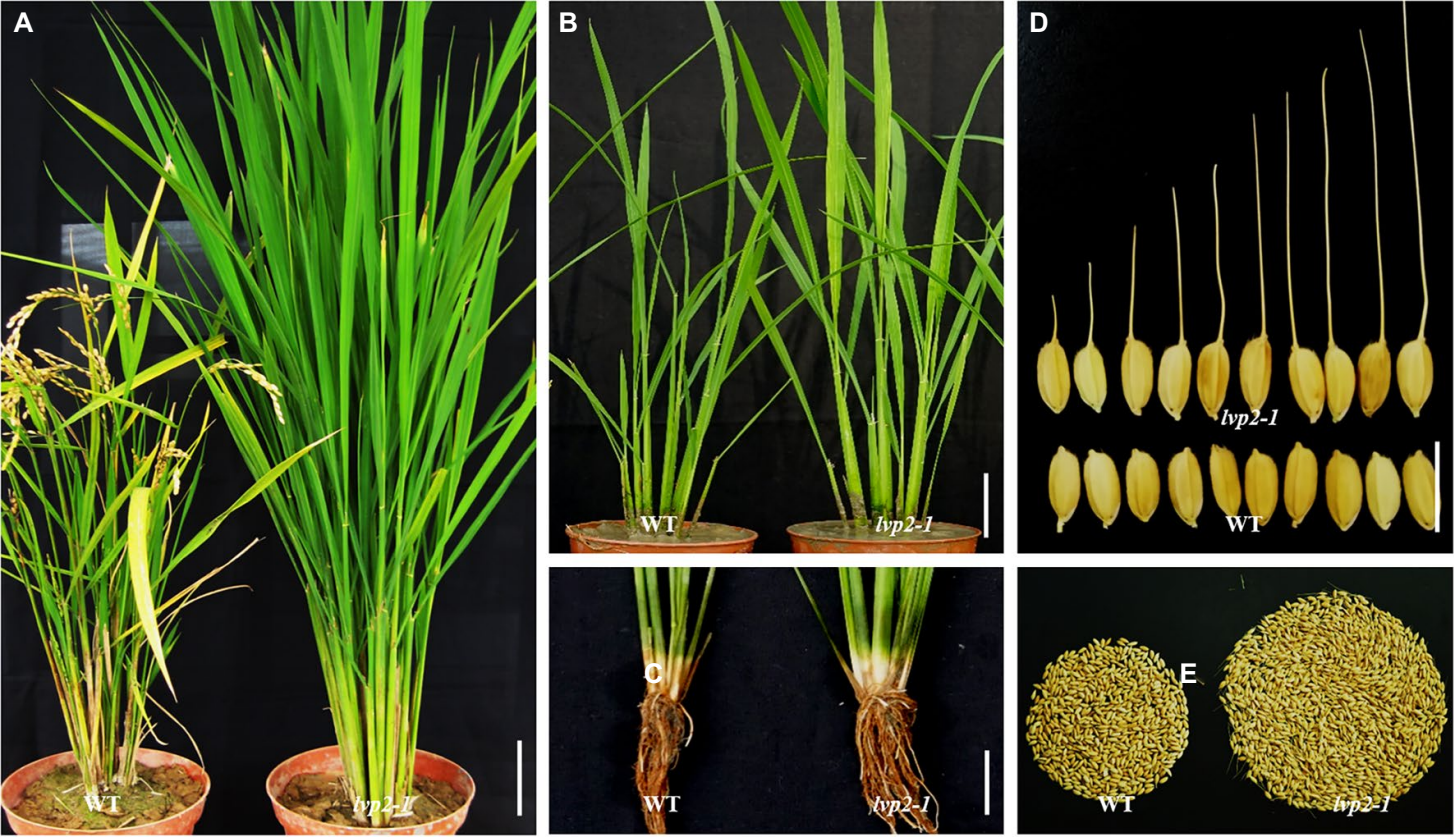
Phenotypes of *lvp2-1* and WT. **(A)** WT and *lvp2-1* under natural day-length condition. **(B,C)** Plants **(B)**, roots and tillers **(C)** of WT and *lvp2-1* at 45 days after sowing under NLD condition. **(D)** Sizes and shapes of WT and *lvp2-1* seeds. **(E)** Seeds per plant of WT and *lvp2-1*. Scale bars = 10 cm in **(A-C)** and 1 cm in **(D)**.

The *lvp2-1* mutant, which displayed a long staying-green period and extremely late flowering similar to that of previously reported *lvp2* mutant (Sun et al., 2012), was identified from the progeny of EMS mutagenesis of Nipponbare, a *japonica* rice variety (Figure 1A). To identify the photoperiod response of *lvp2-1*, we examined the heading date of *lvp2-1* under the natural LD from July to September (NLD, ≥14 h light) condition in Fuzhou (26°08′) and the natural SD from January to March (NSD, ≤10 h light) condition in Sanya (18°10′). The wild type (WT) headed 24.9 days earlier in Sanya (64.6 days) than in Fuzhou (89.5 days; Table 1), whereas the mutant showed extremely late flowering in both Fuzhou (143.4 days) and Sanya (142.5 days), with only about 1 days delay under NLD (Table 1). To confirm this result, we grew *lvp2-1* and WT under both SD (10/14 h light/dark) and LD (14/10 h light/dark) conditions in the growth chamber. The photoperiod response of *lvp2-1* in the growth chamber was completely consistent with that under the natural field conditions (Table 1). These observations indicated that *lvp2-1* was photoperiod-insensitive, suggesting that *LVP2* plays an important role in increasing photoperiod sensitivity and acts as a flowering promoter under all photoperiod conditions in rice.

**Table 1:** Main agronomic traits of WT, *lvp2-1*, and *lvp2-5*.

Traits	WT (Nipponbare)	*lvp2-1*	*lvp2-5*
Heading date (NSD, d)	64.6 ± 0.9	142.5 ± 1.3^**^	142.8 ± 0.7^**^
Heading date (SD, d)	62.9 ± 1.3	141.3 ± 1.7^**^	141.5 ± 1.2^**^
Heading date (NLD, d)	89.5 ± 1.5	143.4 ± 1.1^**^	143.7 ± 1.9^**^
Heading date (LD, d)	91.1 ± 0.6	144.2 ± 0.6^**^	143.5 ± 1.3^**^
Length of flag leaf (cm)	31.28 ± 2.34	59.31 ± 4.49^**^	58.73 ± 3.93^**^
Width of flag leaf (cm)	1.36	1.67^**^	1.65^**^
Leaf number	8–9	18–20^**^	18–20^**^
Culm length (cm)	49.8 ± 3.49	72.68 ± 5.32^**^	73.12 ± 4.75^**^
Tiller/panicle number	8.8	21.8^**^	22.3^**^
Panicle length (cm)	13.93 ± 1.37	14.11 ± 1.13	14.03 ± 1.22
Grain no. per panicle	102.35	104.17	103.82
Kilo-grain weight (g)	20.51	20.72	20.38

lvp2-5, a non-functional allele mutant created by CRISPR/Cas9 editing (Supplementary Figure S1).

** *p ≤ 0.01 in comparison with WT, means ± SD (n > 20)*.

### *lvp2-1* Plant Grew Vigorously With Increased Biomass and Grain Yield

*lvp2-1* exhibited vigorous growth throughout the entire vegetative phase under both SD and LD conditions. We compared several morphological traits of *lvp2-1* with those of WT under the natural day length condition in Fuzhou, including size of leaf, number of leaves per plant, number of tillers per plant, culm length, and so on. This mutant showed similar phenotypes (Table 1). Compared with WT, *lvp2-1* had significantly longer and wider leaves and taller culms (Figures 1A,B; Table 1), and produced much more leaves and tillers (Table 1), but its leaf emergence rate and tiller emergence rate remained almost the same as those of WT (Figures 1B,C). In addition, the mutant generated more lateral roots than WT (Figure 1C), which would possibly enable itself to obtain more nutrition for growth. These observations suggest that *LVP2* controls biomass-driven growth. In the reproductive phase, no remarkable differences were found between *lvp2-1* and WT in panicle length, number of grains per panicle, seed-setting rate, grain size, grain shape, and 1,000-grain weight (Table 1). However, *lvp2-1* produced over twice as many grains as those produced by WT due to its dramatic increase in effective tillers/panicles (Figure 1E; Table 1), suggesting that *lvp2-1* may have the potential to be used for increasing yield in breeding programs. Besides, *LVP2* showed the function of repressing awn development. Most of the *lvp2-1* grains had a long awn, while the WT grains were hardly awned (Figure 1D). In summary, *LVP2* had pleiotropic effects on vegetative growth and reproductive development, and loss of *LVP2* function would dramatically increase the biomass and grain yield of the plant.

### *OsLUX* Mutation Was Responsible for the *lvp2-1* Phenotype

**Figure 2:**
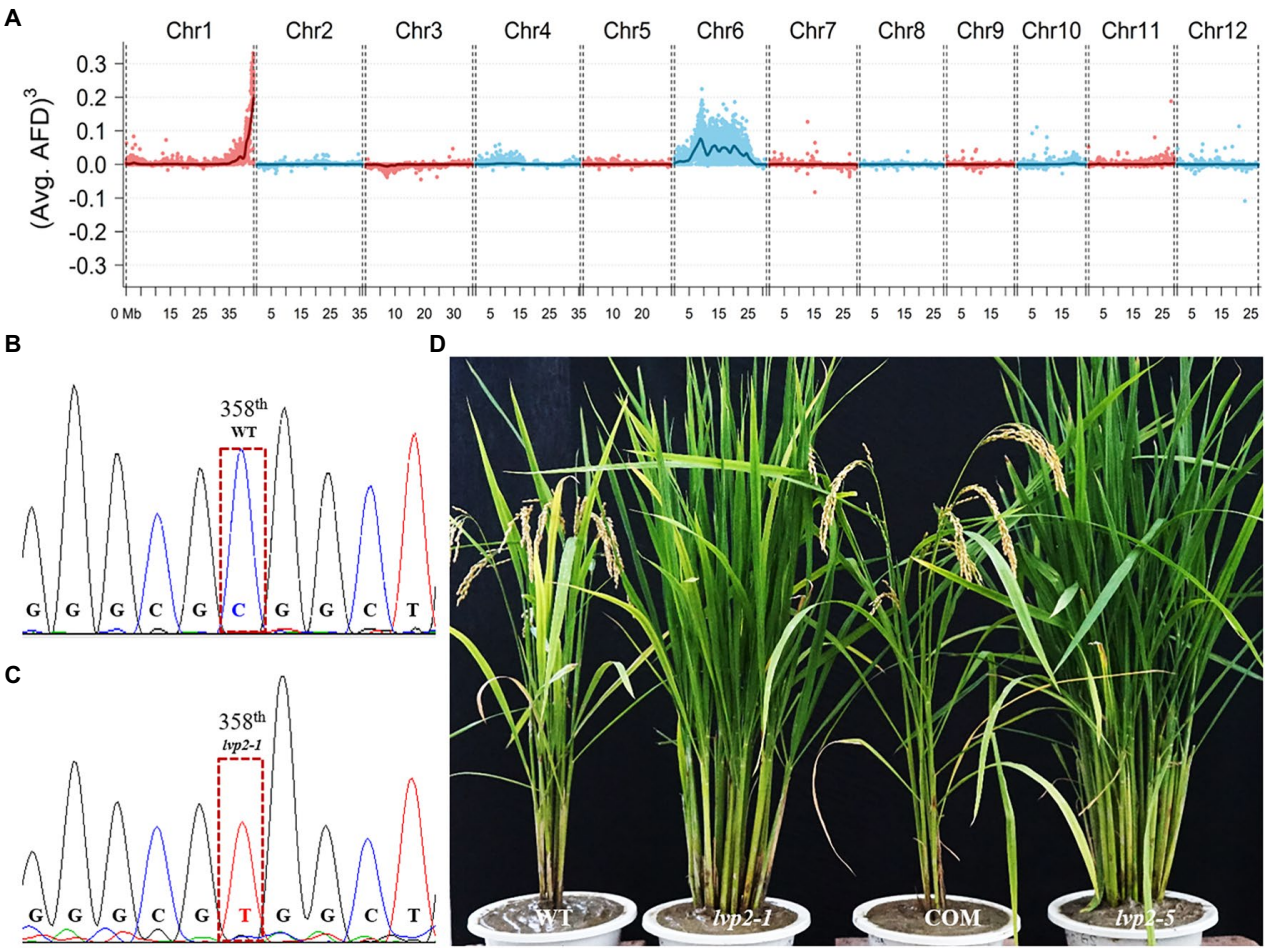
Identification and characterization of *OsLUX* function. **(A)** Mapping and screening the candidate gene of *LVP2* by resequencing the DNA pools of WT and mutant. *LVP2* was located on the terminal region of the long arm of chromosome1. **(B,C)** A single base transversion (C to T) was found in *lvp2-1*, which resulted in the change of the 120th aa from Arginine to Tryptophan. **(D)** Functional validation of *OsLUX via* genetic complementation and gene editing. The WT phenotype was recovered by genetic complementation in the transgenic *lvp2-1* plant, whereas the loss-of-function mutant plant showed *lvp2-1* phenotype. COM, a genetic complementation plant; *lvp2-5*, a non-functional allele generated by CRISPR/Cas9 editing, which encoded a truncated peptide of OsLUX consisting of only 18 amino acids.

Genetic analysis showed that the phenotype of *lvp2-1* was caused by a single gene mutation (Supplementary Table S3). To identify the *LVP2* locus, we crossed *lvp2-1* with variety DZ60 to develop an F_2_ population, and a total of 389 progeny lines (F_2:3_) which display extremely late flowering and other phenotypic characteristics similar to *lvp2-1* were selected for gene mapping. Using the RM-series rice microsatellite markers, we mapped *LVP2* on the terminal region of the long arm of chromosome 1, linked with RM414. To identify the candidate gene, we performed whole-genome resequencing of the DNA pools of WT and mutant, each consisting of 30 lines. Based on the sequence information obtained from resequencing, the *LVP2* locus was also mapped on the terminal region of the long arm of chromosome 1, agreeing with the results of linkage-based mapping (Figure 2A). By comparing the nucleotide sequences of the genomic fragments in this region, we found that there was a single base transition from C (CGG) to T (TGG) in gene *LOC_Os01g74020* (*OsLUX*), which resulted in a change from Arginine to Tryptophan at the 120^th^ amino acid (aa) of OsLUX in *lvp2-1* (Figures 2B,C). OsLUX is a MYB-like transcription factor consisting of 238 aa homologous to *Arabidopsis* LUX (Supplementary Figure S1). The mutation in *lvp2-1* was located within the crucial GARP domain (117^th^–175^th^ aa) in OsLUX (Supplementary Figure S2). The ORF sequences of all the other genes in this region remained unchanged between WT and *lvp2-1*. Expression analysis showed that the rhythm pattern of *OsLUX* expression in *lvp2-1* was almost the same as that in WT, but the expression level in *lvp2-1* was significantly downregulated (Supplementary Figure S3). These results strongly suggested that the *lvp2-1* phenotype was caused by the mutation in *OsLUX*.

To validate the candidate gene, we constructed a complementary plasmid pCAMBIA1300*-OsLUX* carrying a 3,420-bp wild-type genomic DNA, which covered the entire *OsLUX* gene (717-bp) and its upstream promoter region. By introducing the plasmid into *lvp2-1*, the mutant phenotype was rescued and restored to that of WT in all of 25 transgenic lines (Figure 2D; Supplementary Table S4), confirming that *OsLUX* is *LVP2*. To further validate and investigate *OsLUX*, we created the loss-of-function mutants of *OsLUX* from WT Nipponbare using CRISPR/Cas9 technology. Eleven effectively edited lines were obtained, which could be classified into six types, including one-base insertion between the 55^th^ and the 56^th^ bases (Types 1–3, 6 lines), deletion of the 55^th^ base (Type 4, 3 lines), 16-base deletion of 42^nd^-57^th^ base (Type 5, 1 line), and 274-base deletion of 30^th^-303^rd^ base (Type 6, 1 line). We named these lines as *lvp2-2* to *lvp2-7*. All of the edited alleles were predicted to encode a short peptide lacking the crucial GARP domain (Supplementary Table S5). Hence, all of them should be non-functional. As expected, all of the edited lines exhibited the same phenotype as that of *lvp2-1* in photoperiod sensitivity, heading date, and other characteristics under various photoperiod conditions (Table 1; Figure 2D), indicating that *lvp2-1* should be a non-functional allele as well. We also developed *OsLUX* overexpression lines, but the gene expression was not noticeably increased and no significant phenotype alteration was observed in the transgenic lines (Supplementary Figure S4), suggesting that *OsLUX* represses the expression of itself, similar to *LUX* in *Arabidopsis*.

### *OsLUX* Showed a Constitutive Rhythmic Expression Pattern

**Figure 3:**
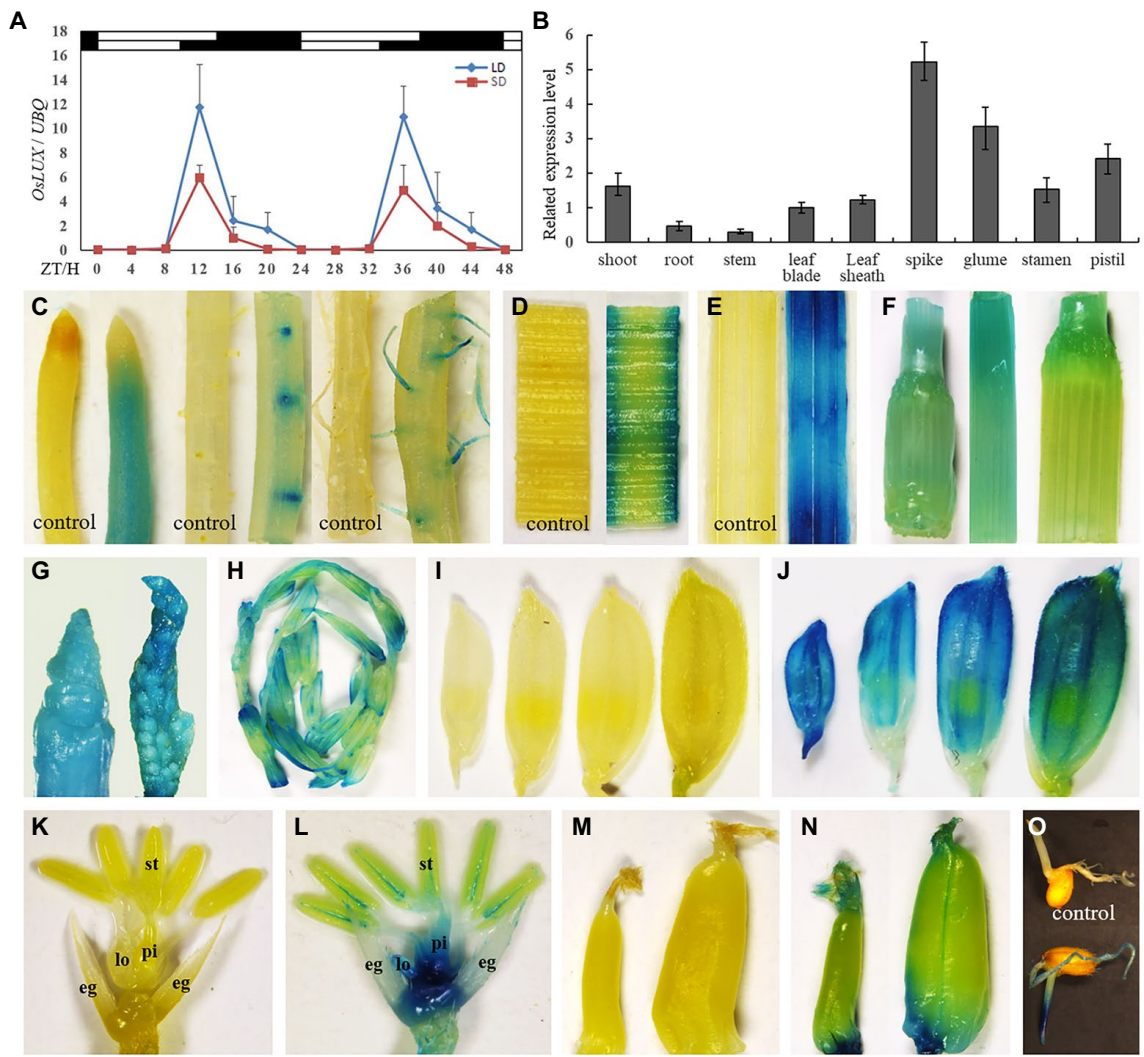
qRT-PCR and GUS analyses of *OsLUX* expression pattern. **(A)** The rhythmic expression of *OsLUX* under both LD and SD conditions in Nipponbare. Rice leaves were collected at the indicated time points from 35- and 70-day-old plants grown under SD and LD, respectively. The expression levels are relative to the *UBQ* mRNA. Values and error bars are the mean and SD of three replicates. The white bars and black bars at the top represent the light and dark periods, respectively. ZT, Zeitgeber time. **(B)** *OsLUX* transcript accumulation in different tissues and organs. Values and error bars are the mean and SD of three replicates. **(C–O)** GUS detection of *OsLUX* transcript accumulation in roots of different parts or developmental stages **(C)**, young leaf blade **(D)** and leaf sheath **(E)**, stems of different parts or developmental stages **(F)**, young panicle **(G,H)**, developing spikelet **(J)**, floral organs with lemma and palea removed **(L)**, developing seed **(N)**, and the germ and radicle of germinating seed (**O)**. **(I)**, **(K)** and **(M)** were controls. eg, extra glums; lo, lodicule; st, stamen; pi, pistil.

To investigate whether the expression of *OsLUX* is rhythmic, we assayed its expression every 4 h in a 48-h time course under both LD (14 h light) and SD (10 h light) conditions. The transcript level of *OsLUX* showed an obvious diurnal circadian rhythm of the daily 24 h cycle, in which it started to accumulate at ZT8, reached the peak approximately at the dusk, and slowly decreased during the night under both LD and SD, but the peak under LD was almost twice as high as that under SD (Figure 3A). To examine whether *OsLUX* expression is organ-specific, quantitative real-time PCR (qRT-PCR) was performed for different organs. *OsLUX* expression was detected in all the organs examined, with the highest accumulation occurring in 5-mm-long young panicle and developing floral organs, followed by shoot and leaf, and weak in shoot, root, and stem (Figure 3B). To confirm the results of qRT-PCR analysis and intuitively display the spatio-temporal expression characteristic of *OsLUX*, we fused the promoter of *OsLUX* with GUS (β-glucuronidase) reporter gene and transferred the fused vector into rice with the mediation of *Agrobaterium tumefaciens*. Totally, 23 transgenic lines were obtained (Supplementary Figure S5). The results of GUS activity examination indicated that *OsLUX* was expressed in all organs including root, stem, leaf, young panicle, spikelet, and developing seed (Figures 3C–O). This was agreement with the results of qRT-PCR. However, GUS analysis indicated that *OsLUX* expression was not uniform within organs. In root, *OsLUX* expression was basically limited to the actively developing young roots and young root hairs, but little in the mature zones (Figure 3C). Developing leaf sheath displayed stronger expression than developing leaf blade (Figures 3D,E). Elongating stem showed higher expression than mature stem (Figure 3F). *OsLUX* expression was strong in developing young panicle and the first internode from top (Figure 3G), but decreased later (Figure 3H). In developing spikelet, *OsLUX* expression was observed in all floral organs, with the strongest in the lemma/palea and receptacle (Figures 3I–L). In addition, *OsLUX* expression was also detected in developing seed (Figures 3M,N) and the germ and radicle of germinating seed (Figure 3O).

### *OsLUX* Regulated Genes Associated With the Circadian Clock

**Figure 4:**
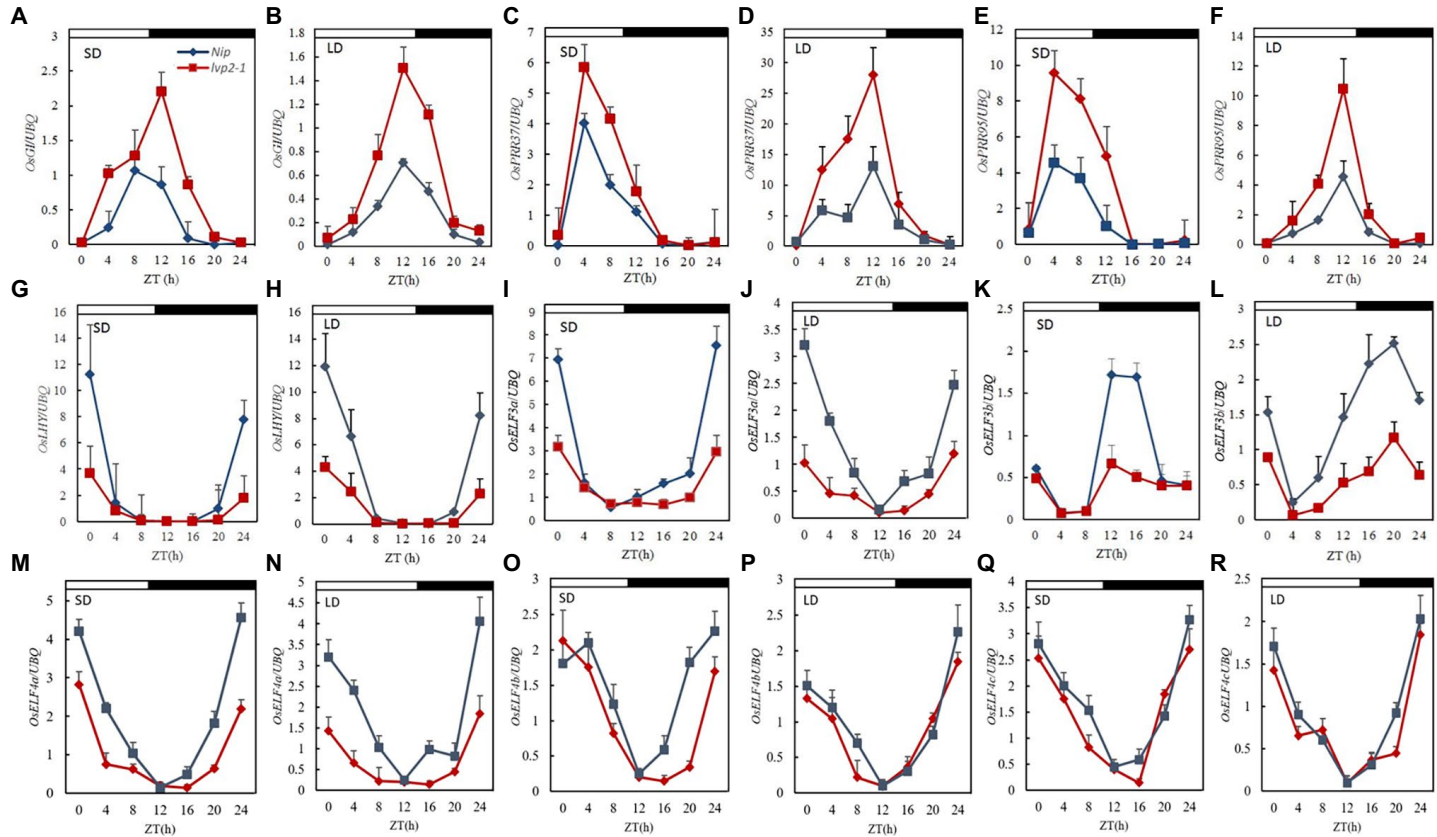
Diurnal expression of clock-associated genes in WT and *lvp2-1* under both SD and LD conditions. Diurnal expression patterns of *OsGI* **(A,B)**, *OsPRR37* **(C,D)**, *OsPRR95* **(E,F)**, *OsLHY* **(G,H)**, *OsELF3a* **(I,J)** *OsELF3b* **(K,L)**, *OsELF4a* **(M,N)**, *OsELF4b* **(O,P)**, and *OsELF4c* **(Q,R)** in Nipponbare (blue diamonds) and *lvp2-1* mutant (red squares) plants in SD and LD detected by qRT-PCR analysis. Rice leaves were collected at the indicated time points from 35- and 70-day-old plants grown under LD (14 h light) and SD (10 h light) conditions, respectively. The expression levels are relative to the *UBQ* mRNA. Values and error bars are the mean and SD of three replicates. The white bars and black bars at the top represent the light and dark periods, respectively. Nip, Nipponbare; ZT, Zeitgeber time.

*Arabidopsis LUX* affects the expression of circadian clock genes. To investigate the effect of *OsLUX* mutation on the circadian clock, we compared the diurnal expression of several known or likely-to-be circadian clock genes in WT and that in *lvp2-1* under both SD and LD, including five with known function (*OsGI*, *OsPRR37*, *OsLHY*, *OsELF3a*, and *OsELF3b*), one unidentified function (*OsPRR95*), and three speculative *OsELF4* genes (*OsELF4a*, *OsELF4b*, and *OsELF4c*; Supplementary Figure S6). The rhythmic expression patterns of these genes in *lvp2-1* were similar to those in WT under both SD and LD conditions, but the expression levels of the most genes changed (Figure 4). Among them, *OsGI*, *OsPRR37*, and *OsPRR95* showed higher expression peaks in *lvp2-1* than in WT (Figures 4A–F), suggesting that *OsLUX* negatively regulates the expression of these genes under both day-length conditions, whereas the expression of *OsLHY*, *OsELF3a*, *OsELF3b*, and *OsELF4a* were dramatically reduced in *lvp2-1* mutant (Figures 4G–N), suggesting that *OsLUX* positively regulates the expression of these genes under both day-length conditions. However, *OsELF4b* and *OsELF4c* did not show significant change under SD and LD conditions (Figures 4O–R). These results suggest that *OsLUX* is essential to the circadian function through positive or negative regulation of the expression of genes associated with the circadian clock in rice.

### *OsLUX* Affected the Expression of Floral Integrator Genes

**Figure 5:**
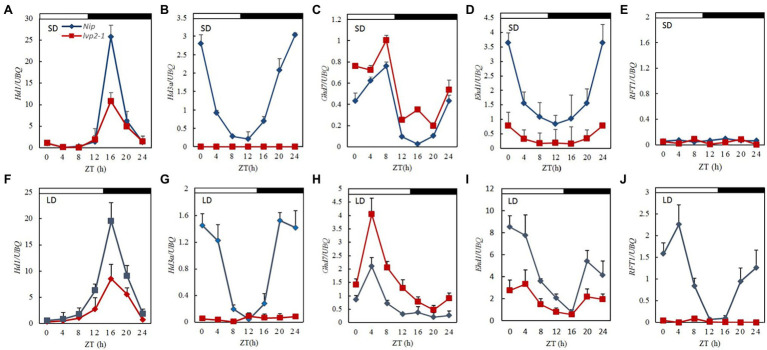
Diurnal expression of photoperiodic flowering pathway genes in *lvp2-1* mutant under both SD and LD conditions. Diurnal expression patterns of *Hd1* **(A,F)**, *Hd3a* **(B,G)**, *Ghd7* **(C,H)**, *Ehd1* **(D,I)** and *RFT1* **(E,J)** in Nipponbare (blue diamonds) and *lvp2-1* (red squares) plants under LD (14 h light) and SD (10 h light) detected by qRT-PCR analysis. Rice leaves were collected at the indicated time points from 35- and 70-day-old plants grown under SD and LD conditions, respectively. The expression levels are relative to the *UBQ* mRNA. Values and error bars are the mean and SD of three replicates. The white bars and black bars at the top represent the light and dark periods, respectively. ZT means Zeitgeber time.

In rice, *Ehd1* and *Hd1* determine the degree of expression of florigenic genes *Hd3a* and *RFT1* through distinct pathways under a given photoperiod. To identify potential downstream flowering time genes regulated by *OsLUX*, we examined the diurnal expression of five photoperiod flowering-related integrator genes, *Hd1*, *Ghd7*, *Ehd1*, *Hd3a*, and *RFT1*, under both SD and LD. Under SD, the peak of *Hd1* expression was lower in *lvp2-1* than in WT (Figure 5A), and almost no *Hd3a* expression could be detected in *lvp2-1* (Figure 5B). This was likely to be directly responsible for the later flowering of *lvp2-1* under SD. Meanwhile, the peak of *Ghd7* expression was higher in *lvp2-1* than in WT (Figure 5C), suggesting that *OsLUX* represses *Ghd7* expression; while the peak of *Ehd1* expression was lower in *lvp2-1* than in WT (Figure 5D), due to the repression of *Ghd7*. As expected, the expression of these genes except *RFT1* under LD (Figures 5E,J) showed similar alternation patterns to those under SD in *lvp2-1* (Figures 5F–I), consistent with the mutant phenotype of photoperiod-insensitive late flowering (Figure 1). Under SD, very low *RFT1* expression was detected in both WT and *lvp2-1* (Figure 5E), whereas under LD, the peak of *RFT1* expression was markedly lower in *lvp2-1* than in WT (Figure 5J), which led to late flowering in *lvp2-1*. Taken together, *OsLUX* is essential for the photoperiodic control of rice flowering. *OsLUX* functions as a floral promoter by promoting *Hd1* and *Ehd1* expression and repressing *Ghd7* expression under both SD and LD conditions, so as to regulate the expression of downstream florigenic genes *Hd3a* and *RFT1*.

### OsLUX Located in Nucleus and Formed ECs With OsELF4a and OsELF3a or OsELF3b

To investigate the intracellular location of OsLUX, we constructed an expressional vector of the fusion gene of *GFP* (green fluorescent protein) and *OsLUX* to transform rice protoplasts. The green fluorescent signal was observed specifically in nucleus (Supplementary Figure S7), suggesting that OsLUX was anchored in nucleus.

In rice, two *ELF3* orthologs, *OsELF3-1/OsELF3a* and *OsELF3-2/OsELF3b*, have been identified, but no apparent *ELF4* ortholog has been found (Murakami et al., 2007). To identify the putative rice ELF4, we used the full-length sequence of ELF4 to perform BLAST analyses. Four *ELF4* homologues were found from the rice genome sequence, namely, LOC_Os11g40610, LOC_Os03g29680, LOC_Os08g27860, and LOC_Os08g27870, which showed considerable similarities to ELF4 in amino acid sequence. Protein sequence alignment and phylogenetic analysis showed that these ELF4 homologues contained a function-unknown evolutionarily conserved domain DUF1313 (Supplementary Figure S6; Kolmos et al., 2010; Lin et al., 2019). Among these genes, LOC_Os08g27870 transcript was undetectable in rice leaf in this study. Hence, we named LOC_Os11g40610, LOC_Os03g29680 and LOC_Os08g27860 as *OsELF4a*, *OsELF4b* and *OsELF4c*, respectively.

**Figure 6:**
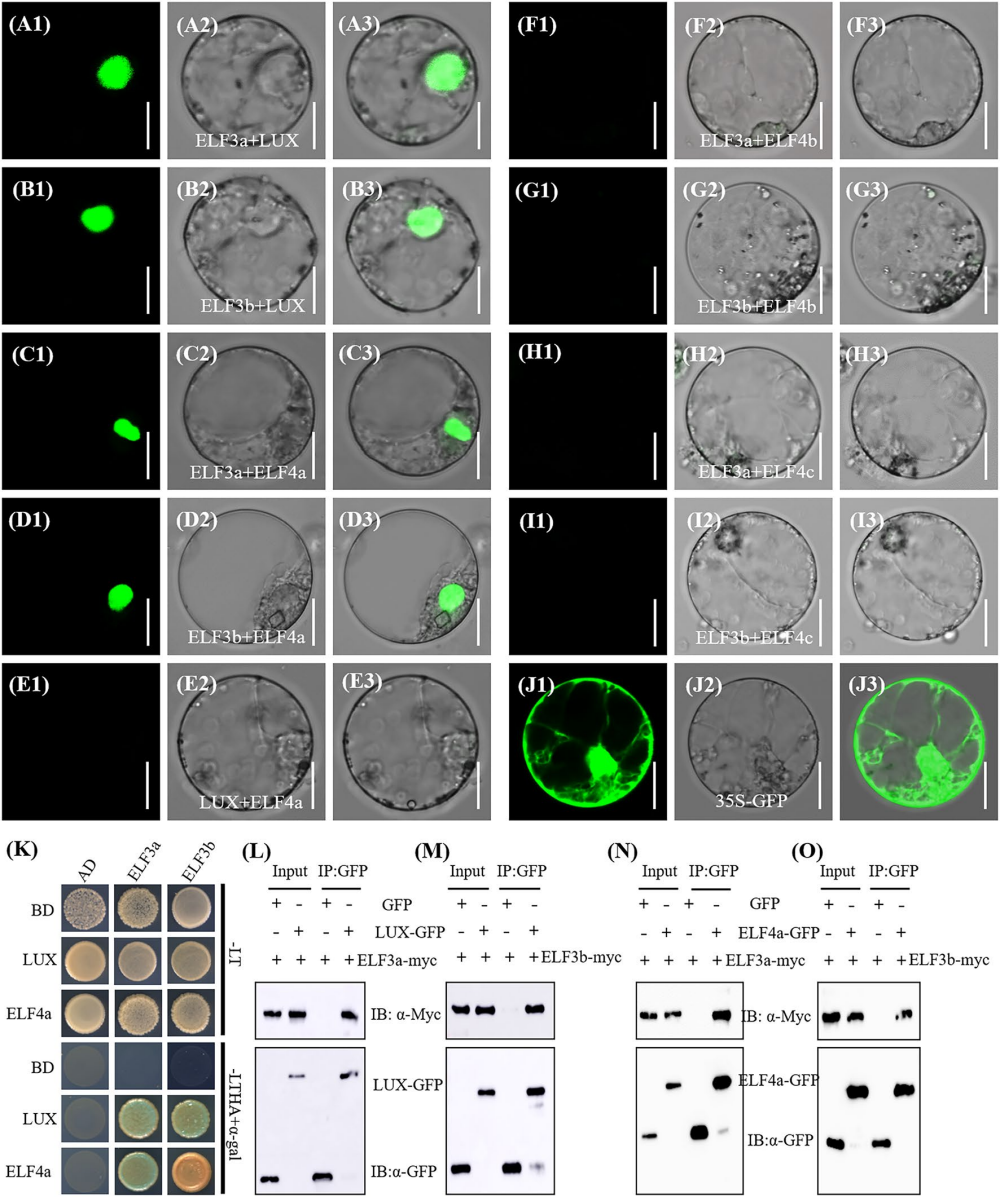
Detection of interactions among **OsLUX,** OsELF3s and OsELF4s. **(A-I)** Bimolecular fluorescence complementation (BiFC) assay of the interaction between OsELF3a and OsLUX **(A)**, OsELF3b and OsLUX **(B)**, OsELF3a and OsELF4a **(C)**, OsELF3b and OsELF4a **(D)**, OsLUX and OsELF4a **(E)**, OsELF3a and OsELF4b **(F)**, OsELF3b and OsELF4b **(G)**, OsELF3a and OsELF4c **(H)**, and OsELF3b and OsELF4c **(I)**. **(J)** Ubi-GFP as the control. (A1-J1) GFP filter; (A2-J2) bright field; (A3-J3) merged images. Scale bars = 10 μm. (K) yeast two-hybrid assay the interactions between LUX or ELF4a with ELF3a or ELF3b, respectively. Transformed yeast cells were grown on non-selective SD media (−Trp-Leu/−LT) and selective SD media (−Trp-Leu-His-Ade/-LTHA) with X-α-Gal. (L-O) Co-IP analysis confirms that OsLUX interacts with OsELF3a **(L)** and OsELF3b **(M)**, and OsELF4a interacts with OsELF3a **(N)** and OsELF3b **(O)**, respectively.

To investigate the components of EC in rice, we performed bimolecular fluorescence complementation (BiFC) assay to test the interactions of OsLUX with two OsELF3s and three OsELF4s. The coding sequences of *OsLUX*, *OsELF3a*, *OsELF3b*, *OsELF4a*, *OsELF4b*, and *OsELF4c* were fused into the N- and/or C-terminal of vectors pSPYNE and pSPYCE, respectively. No fluorescence was observed in the mixed carrier of all the combinations of NE-LUX+CE, NE + CE-LUX, NE-ELF3a + CE, NE-ELF3b + CE, NE + CE-ELF4a, NE + CE-ELF4b, and NE + CE-ELF4c (data not shown). Green fluorescence was observed from the nuclei of rice protoplasts co-transformed with NE-OsELF3a + CE-OsLUX (Figure 6A), NE-OsELF3b + CE-OsLUX (Figure 6B), NE-OsELF3a + CE-OsELF4a (Figure 6C), and NE-OsELF3b + CE-OsELF4a (Figure 6D). However, no green fluorescence was observed using the mixed carrier of NE-OsLUX+CE-OsELF4a (Figure 6E), NE-OsELF3a + CE-OsELF4b (Figure 6F), NE-OsELF3b + CE-OsELF4b (Figure 6G), NE-OsELF3a + CE-OsELF4c (Figure 6H), and NE-OsELF3b + CE-OsELF4c (Figure 6I). The interactions between OsLUX and OsELF3a, OsLUX and OsELF3b, OsELF4a and OsELF3a, and OsELF4a and OsELF3b were further confirmed by yeast two-hybrid assay (Figure 6K) and *in vivo* Co-IP assay (Figures 6L–O). These results indicated that OsLUX forms two ECs with OsELF4a and OsELF3a or OsELF3b, respectively; but OsELF4b and OsELF4c cannot form EC with OsLUX and OsELF3a or OsELF3b. We named the two ECs as LUX-ELF3a-ELF4a and LUX-ELF3b-ELF4a in rice.

### Loss of *OsELF4a* Function Delayed Flowering Time Under LD Condition

In *Arabidopsis*, disruption of *ELF4* results in early flowering and attenuation of rhythmicity, while overexpression of *ELF4* delays flowering (Doyle et al., 2002; Kikis et al., 2005). In addition, two ELF4-Like genes, *EFL1* and *EFL3*, are involved in flowering time regulation. Overexpression of *EFL1* in *elf4* shows normal rhythmicity and delays flowering; overexpression of *EFL3* in *elf4* partially rescues the early flowering phenotype of *elf4* (Lin et al., 2019). To characterize the function of *OsELF4a*, we created the loss-of-function mutants of *OsELF4a* from Nipponbare (WT) using the CRISPR/Cas9 method. In total, five transgenic lines with effective editing in the ORF of *OsELF4a* were obtained (Supplementary Table S6), which could be classified into two types. Four lines harbored a 44-base deletion from 107^th^ to 150^th^, and one line harbored a 43-base deletion from 107^th^ to 149^th^. The two types of deletions were both predicted to encode a truncated short polypeptide consisting of 35 aa plus a missense sequence, leading to complete loss of the conserved DUF1313 domain (Supplementary Figure S6, Supplementary Table S6). Thus, both of them should be null alleles of *OsELF4a*. There was no significant phenotypic difference between these two types of edited lines. We named them *Oself4a-1* and *Oself4a-2*, respectively.

**Figure 7:**
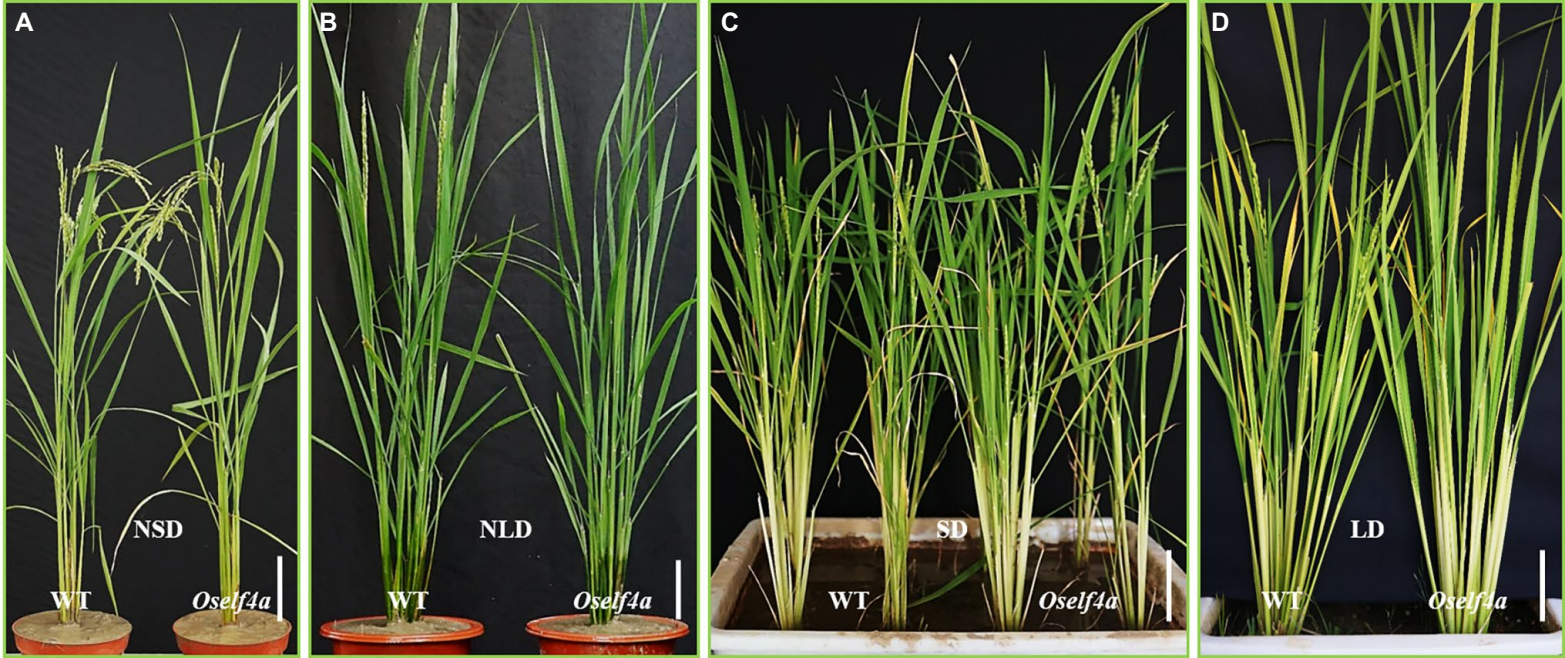
*Oself4a* and WT plants grown under different day-length conditions. **(A,B)** in the field; **(C,D)** in the growth chamber. Scale bar = 10 cm.

To investigate whether *OsELF4a* is involved in flowering time regulation, we planted *Oself4a* mutants and examined their heading dates under the NLD of summer in Fuzhou and the NSD of winter in Sanya. The *Oself4a* mutants showed a marginal (1 or 2 days of) delay in flowering under NSD (Figure 7A), but much greater delay (9 ± 0.45 days, *n* > 30) under NLD compared with WT (Figure 7B). Other main agronomic traits all remained unchanged between WT and the *Oself4a* mutants under both NSD and NLD (Figures 7A,B). To confirm this result, we grew the *Oself4* mutants and WT under SD (10/14 h light/dark) and LD (14/10 h light/dark) conditions in the growth chamber. The results agreed with those under the natural field conditions (Figures 7C,D). These observations reveal that *OsELF4a* functions as a floral promoter under LD condition.

## Discussions

**Figure 8:**
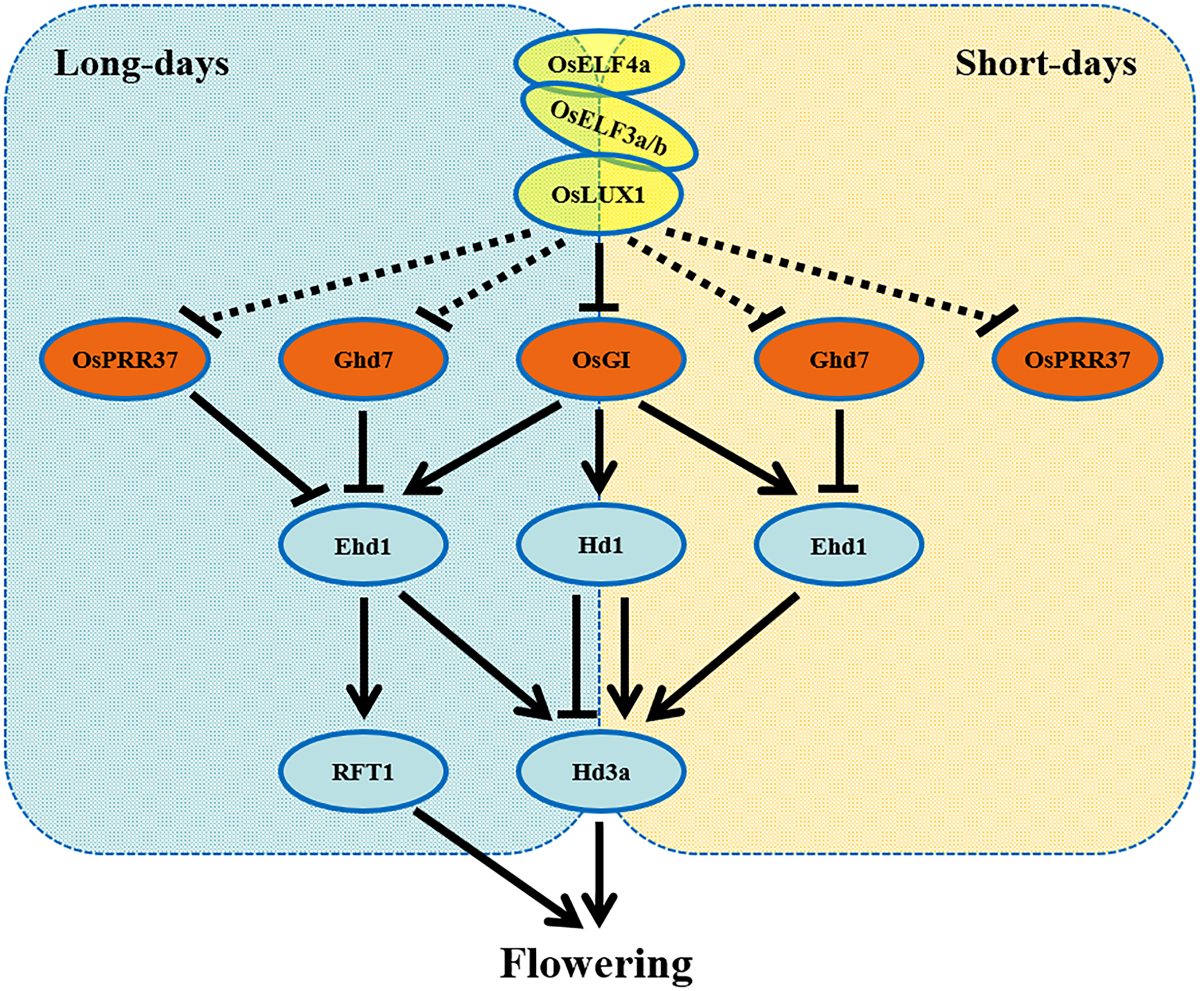
A model for EC-mediated photoperiodic control of flowering in rice. The EC, composed of OsLUX, OsELF3a or OsELF3b, and OsELF4a, is an integrant of repressing flowering in rice. EC controls photoperiodic flowering *via* repressing the expression of *PRR37*, *OsGI*, and *Ghd7* under both LD and SD conditions, so as to regulate the expression of the critical output integrators *Hd1*, *Ehd1*, *Hd3a*, and *RFT1* in rice.

In this study, we identified rice EC composition and characterized its two components OsLUX and OsELF4a. *OsLUX* is the sole rice *LUX* ortholog and is critical for regulating heading date. Disruption *OsLUX* extremely extended the vegetative phase and caused photoperiod-insensitive late flowering and significantly higher grain yield (Figure 1). OsLUX interacted with OsELF4a and OsELF3a or OsELF3b to form two ternary repressive complexes (Figure 6). Unlike *OsLUX* and *ELF3a*, *OsELF4a* functioned as a floral promoter only under LD condition (Figure 7). Our results indicate that the EC genes in rice share the function of promoting flowering. This is agreement with the orthologs of short-day plants, but opposite to the counterparts of long-day species. Taken together, it is likely that rice EC genes display similar but not identical function in controlling flowering time by cooperatively and independently regulating the expression of genes associated with the circadian clock and the output integrators of photoperiodic flowering genes (Figure 8). These findings facilitate our understanding of photoperiodic flowering in plants, especially the short-day crops. We have noticed that when we are preparing this manuscript, a paper of an independent study on the rice EC role in salt tolerance and heading regulation has recently been accepted for publication, in which the findings about the EC composition and the *OELF4a* role in heading date are almost identical to ours, but those about the function of *OsLUX* in heading date are significantly different from ours, which may be due to the weak allele used in their study, in which a T-DNA was inserted upstream of its transcription initiation site and reduced the expression of *OsLUX*, resulting in a slightly delay in heading date under SD condition, but not under LD condition (Wang et al., 2021; in press).

### EC Genes Show Conserved and Divergent Roles Across Species

The EC components LUX, ELF3, and ELF4 can be found in the genomes of land plant lineages, and increasing evidence has shown that EC genes function in flowering time. In *Arabidopsis*, EC members share a similar expression pattern and almost conserved molecular function. Disruption of any of its components leads to common phenotypic defects, displaying arrhythmic circadian period, long hypocotyl, and photoperiod-insensitive early flowering (Nusinow et al., 2011). Meanwhile, overexpression of either *ELF3* or *LUX* complements the *elf4* mutant phenotype (Herrero et al., 2012). These cross-gene complementation in *Arabidopsis* reveals an identical functions of EC genes. Interestingly, cross-species complementation for *Arabidopsis* mutants have also been observed in EC gene orthologs of other plants. For example, both the *ELF3* orthologs from monocot *Brachypodium* and *Setaria* (Huang et al., 2017) and those from dicot LD plant pea (Weller et al., 2012) and SD plant soybean (Lu et al., 2017) can rescue *Arabidopsis elf3* phenotype. Similar to *ELF3*, soybean *LUX2* can complement *Arabidopsis lux* mutants as well (Liew et al., 2017; Fang et al., 2021). These cross-species complementation between monocots and dicots, and LD and SD plants reveal a widely conserved function in EC genes across plant species.

In addition, the potential divergent roles of EC genes have been observed across species as well. In rice, four EC gene orthologs have been characterized. We have seen that *OsLUX*, *OsELF3a/b*, and *OsELF4a* are essential for promoting photoperiodic flowering, but their mutant phenotypes in flowering time and photoperiodic response are significantly different (Figures 1, 7; Saito et al., 2012; Zhao et al., 2012; Yang et al., 2013; Wang et al., 2021). Apart from affecting photoperiodic flowering, *OsLUX* also showed profound effects on the global regulation of growth and development in this study. The non-functional alleles altered some pivotal agronomic traits, displaying extremely long juvenile phase and vigorous biomass-driven growth, which led to producing more leaves, tillers, and lateral roots (Figures 1, 2E; Table 1) and directly contributed to the high biomass and grain yield (Figure 1E). *OsELF3a* has pleiotropic effects as well, but its mutation mainly influences the traits of panicle number and panicle length (Saito et al., 2012), which are markedly different from those of *lvp2* (Figures 1, 2; Table 1). Disruption of *OsELF4* did not cause significant changes in other traits in all day length. These observations showed that, unlike the case in *Arabidopsis*, the fundamental functions of the four rice EC genes, *OsLUX*, *OsELF4*, *OsELF3*a/*3b*, are not completely consistent with each other in most agronomic characters including flowering time.

Besides, we have also noticed that EC genes have inhibiting effect on flowering in LD species, such as pea, lentil, wheat, and barley. All mutant alleles of EC gene orthologs identified in these species show early flowering regardless of day length, resembling the mutant alleles of *LUX*, *ELF3*, and *ELF4* in *Arabidopsis* (Liew et al., 2009; Faure et al., 2012; Mizuno et al., 2012; Weller et al., 2012; Zakhrabekova et al., 2012; Campoli et al., 2013; Gawroński et al., 2014; Boden et al., 2014; Liew et al., 2014; Mizuno et al., 2016). However, all mutant alleles of *LUX*, *ELF3*, and *ELF4* orthologs identified so far in SD crops, such as rice and soybean, have later flowering time than wild-type lines (Lu et al., 2017; Bu et al., 2021; Fang et al., 2021; Wang et al., 2021; Figure 7), implying that EC genes promote flowering in SD plants, opposite to the counterparts in LD plants. The functional difference of EC components in SD plants and LD plants probably resides in the regulation or activity of downstream integrator components, rather than in the EC gene itself. This speculation, however, needs further experimental verification.

### The Possible Pathway for EC’s Promoting Flowering in Rice

Photoperiodic flowering in plant is closely tied to EC, a core component of circadian clock. In *Arabidopsis*, EC plays a vital role in circadian rhythms and flowering. As an integrant of repressing flowering, EC represses the expression of circadian genes *PRR7*, *PRR9*, and *GI* directly and of output genes, such as *CO*, *FLC*, and *FT* indirectly, so as to control flowering time (Nusinow et al., 2011; Chow et al., 2012; Mizuno et al., 2014; Song et al., 2015; Silva et al., 2020). Unlike the case in *Arabidopsis*, where only one FT presents, there are two florigens in rice, Hd3a and RFT1, and thus two pathways, including the conserved *OsGI*-*Hd1*-*Hd3a* pathway (similar to the *Arabidopsis GI*-*CO*-*FT*) and a unique *Ghd7*-*Ehd1*-*Hd3a*/*RFT1* pathway, have evolved to adapt to the photoperiodic flowering under SD or LD condition. Each pathway is regulated by the coincidence of the internal circadian clock and the external photoperiodic information (Song et al., 2015).

In rice, OsLUX interacts with OsELF4a and OsELF3a or OsELF3b to form two ECs (Figure 6). Nevertheless, the interaction between OsELF3a and OsLUX is stronger than that between OsELF3b and OsLUX (Wang et al., 2021). Two *ELF3* orthologs are likely simultaneously required for the control of flowering time, but *OsELF3a* plays a more predominant role (Saito et al., 2012; Zhao et al., 2012; Yang et al., 2013), while the contribution of *OsELF3b* to flowering time remains controversy (Fu et al., 2009; Zhao et al., 2012). These findings suggest that the OsLUX-OsELF3a-OsELF4a is likely the dominant promoter for photoperiodic flowering.

We found that *OsLUX* participated the regulation of rice photoperiodic flowering *via* coordinating the expression of genes associated with the circadian clock and the output integrators under both LD and SD conditions. As a transcriptional repressor, OsLUX suppressed *Ghd7* expression, while its non-functional mutation elevated the expression of *Ghd7* under both LD and SD, leading to reduced expression of *Ehd1*, *Hd3a*, and *RFT1* (Figures 4, 5). Besides, *OsLUX* also negatively regulated the expression of *OsGI* (Figures 4A,B) and *OsPRR37* (Figures 4C,D), of which the former is responsible for *Hd1* and *Ehd1* expression (Hayama et al., 2003; Itoh et al., 2010), and the latter preferentially suppresses the expression of *Ehd1* but not *Hd1*, making the expression of *Hd3a* and *RFT1* reduced under LD, but not under SD (Gao et al., 2014; Liu et al., 2018). Interestingly, similar to that of OsLUX, OsELF3a also acts as a flowering activator under both LD and SD conditions, and disruption of *OsELF3a* function causes similar expression alteration of these genes associated with photoperiodic flowering under various conditions (Saito et al., 2012; Zhao et al., 2012). Nevertheless, OsELF4a displays a regulatory mechanism similar to those of OsLUX and OsELF3a under SD condition, but a more complicated regulatory mechanism under LD condition (Wang et al., 2021). In addition, *Arabidopsis* ELF4-like proteins do not appear in EC, but influence flowering time (Lin et al., 2019). Therefore, it will be of interest to further investigate the roles of ELF4-like genes in rice heading.

Taken together, as an integrant, OsLUX, OsELF3a, and OsELF4a promote rice flowering through the same pathway. It is likely that they regulate the expression of genes associated with photoperiodic flowering cooperatively and independently, similar to soybean GmLUX2 and GmELF3a (Fang et al., 2021). Based on previous findings (Wei et al., 2020; Wang et al., 2021) and our results, we draw a simple outline model to explain how EC regulates photoperiodic flowering in rice (Figure 8). As a complex of transcriptional repressor, EC acts at the upstream of *PRR37* and *Ghd7* (genetically) and *OsGI* (physically), repressing the expression of these genes under both LD and SD (Figures 4, 5; Wang et al., 2021), so as to regulate the expression of *Ehd1* and *Hd1*. *Ehd1* and *Hd1* determine the expression level of florigenic genes *Hd3a* and *RFT1* through distinct pathways under a given photoperiod. *Hd1* is crucial for SD photoperiodic induction of *Hd3a* (Izawa, 2007). *Ehd1* promotes flowering independent of *Hd1* under SD, but also promotes flowering under LD when *Hd1* represses *Hd3a* expression, whereas *Ghd7* negatively regulates *Ehd1* expression (Woods et al., 2014).

## Data Availability Statement

The original contributions presented in the study are included in the article/Supplementary Material, further inquiries can be directed to the corresponding authors.

## Author Contributions

YD supervised the project. YD and WW conceived and designed the research plans. YD, ZC, YZ, WT, XC, CL, YL, and YY performed the experiments and collected and analyzed the data. YD and WW analyzed the data wrote the manuscript. All authors contributed to the article and approved the submitted version.

## Funding

This work was supported by grants from the National Natural Science Foundation of China (No. 31871600), Sci-Tech Innovation Special Fund of Fujian Agriculture and Forestry University (No. CXZX2018112 and CXZX2016160), the Natural Science Foundation of Fujian Province (No. 2021J01074), and Chinese Scholarship Council (201908350032).

## Conflict of Interest

The authors declare that the research was conducted in the absence of any commercial or financial relationships that could be construed as a potential conflict of interest.

## Publisher’s Note

All claims expressed in this article are solely those of the authors and do not necessarily represent those of their affiliated organizations, or those of the publisher, the editors and the reviewers. Any product that may be evaluated in this article, or claim that may be made by its manufacturer, is not guaranteed or endorsed by the publisher.
