# Supplementary material for: LUX ARRHYTHMO Interacts With ELF3a and ELF4a to Coordinate Vegetative Growth and Photoperiodic Flowering in Rice
Source: Front Plant Sci. 2022 Mar 17;13:853042. doi: 10.3389/fpls.2022.853042 (PMC8993510; doi:10.3389/fpls.2022.853042)
Supplement: Supplementary file 1 [file Data_Sheet_1.docx]

**Supporting information**

The following materials are available in the online version of this article.


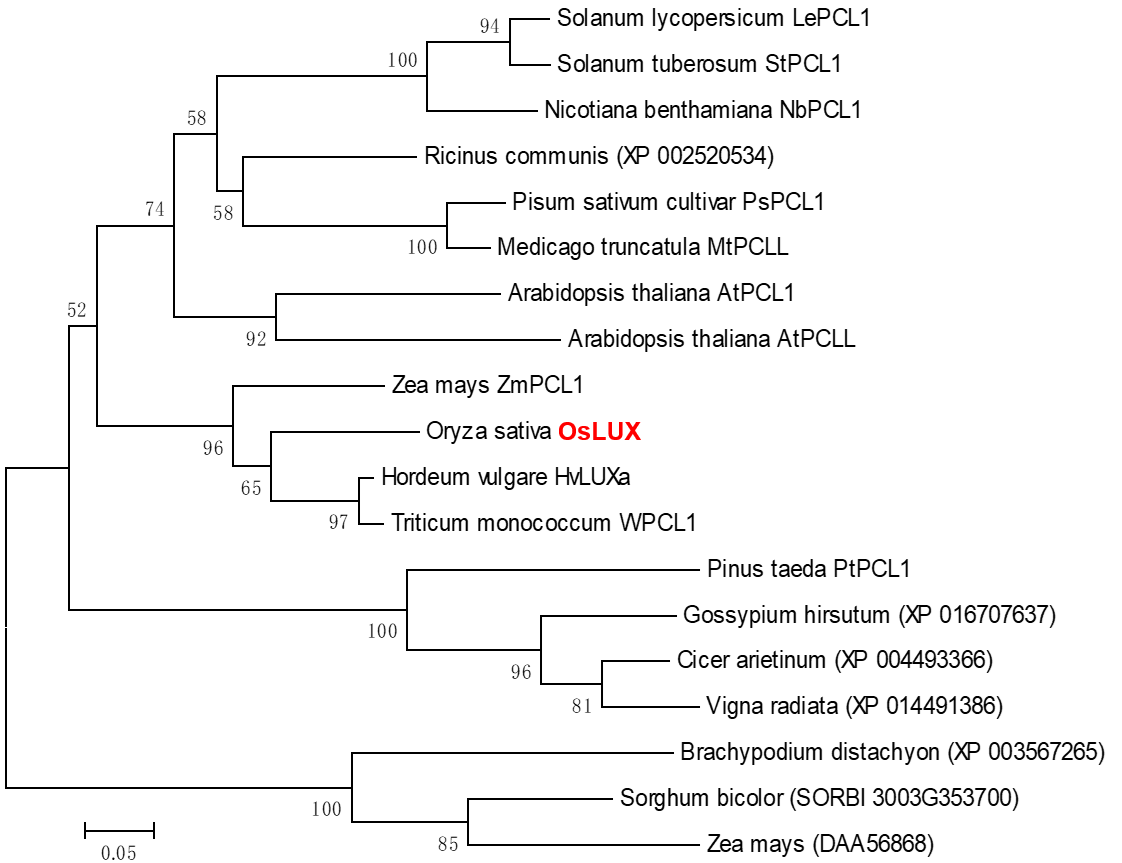


**Figure S1 Phylogenetic tree of LUX/PCL1 proteins from rice, *Arabidopsis* and other species.**


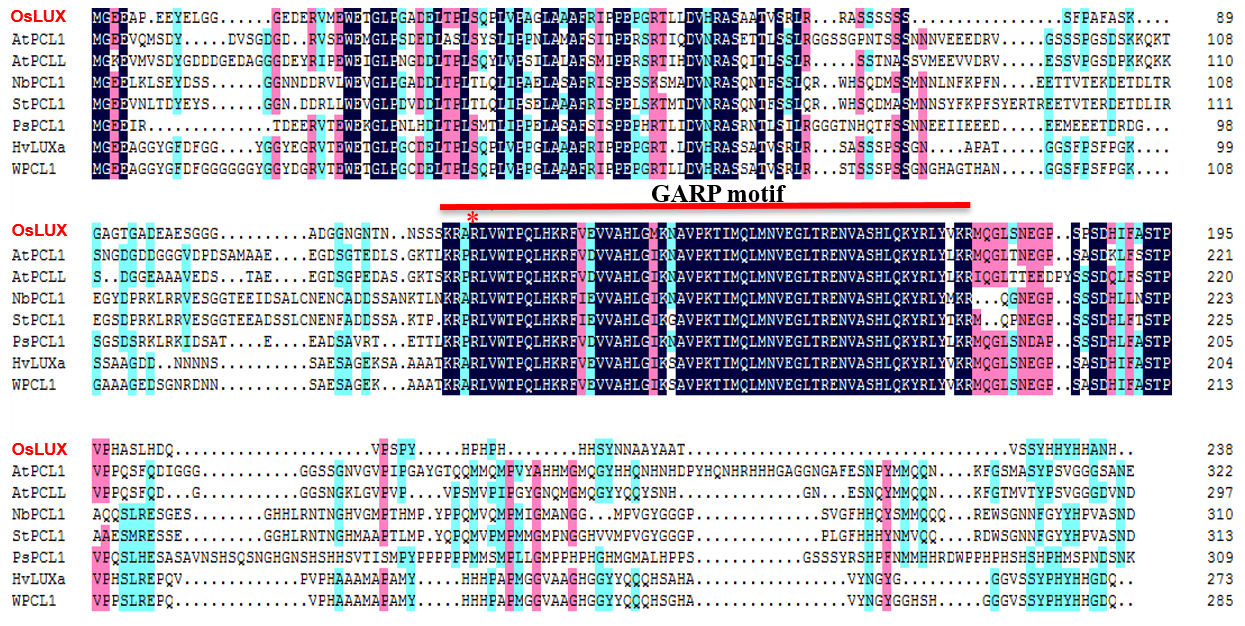


**Figure S2 Homology analysis of plant LUX/PCL1 proteins and the functional domains.**

All LUX/PCL1 proteins contain a conservative GARP motif in plants. Red asterisk indicates the mutation site of *lvp2-1* in the GARP domain (117^th^–175^th^ aa).


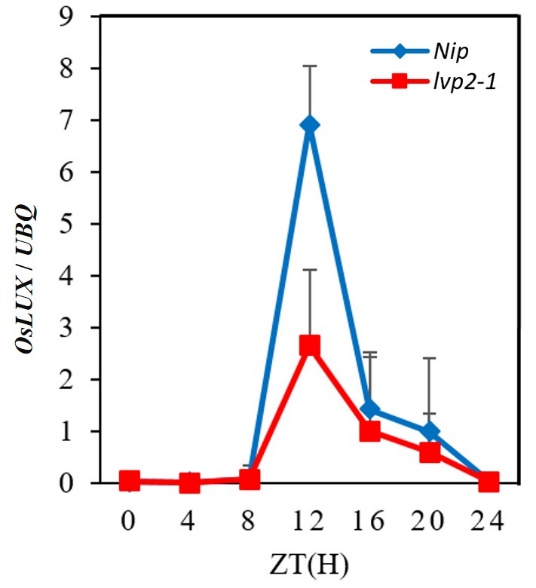


**Figure S3** **qRT-PCR detection of *OsLUX* expression level in WT and *lvp2-1* mutant** **under NLD.**

Rice leaves were collected at the indicated time points from 70-day-old plants grown under natural LD. The expression levels are relative to the *UBQ* mRNA. Values and error bars are mean and standard deviation of three replicates. Nip, Nipponbare; ZT, Zeitgeber time.


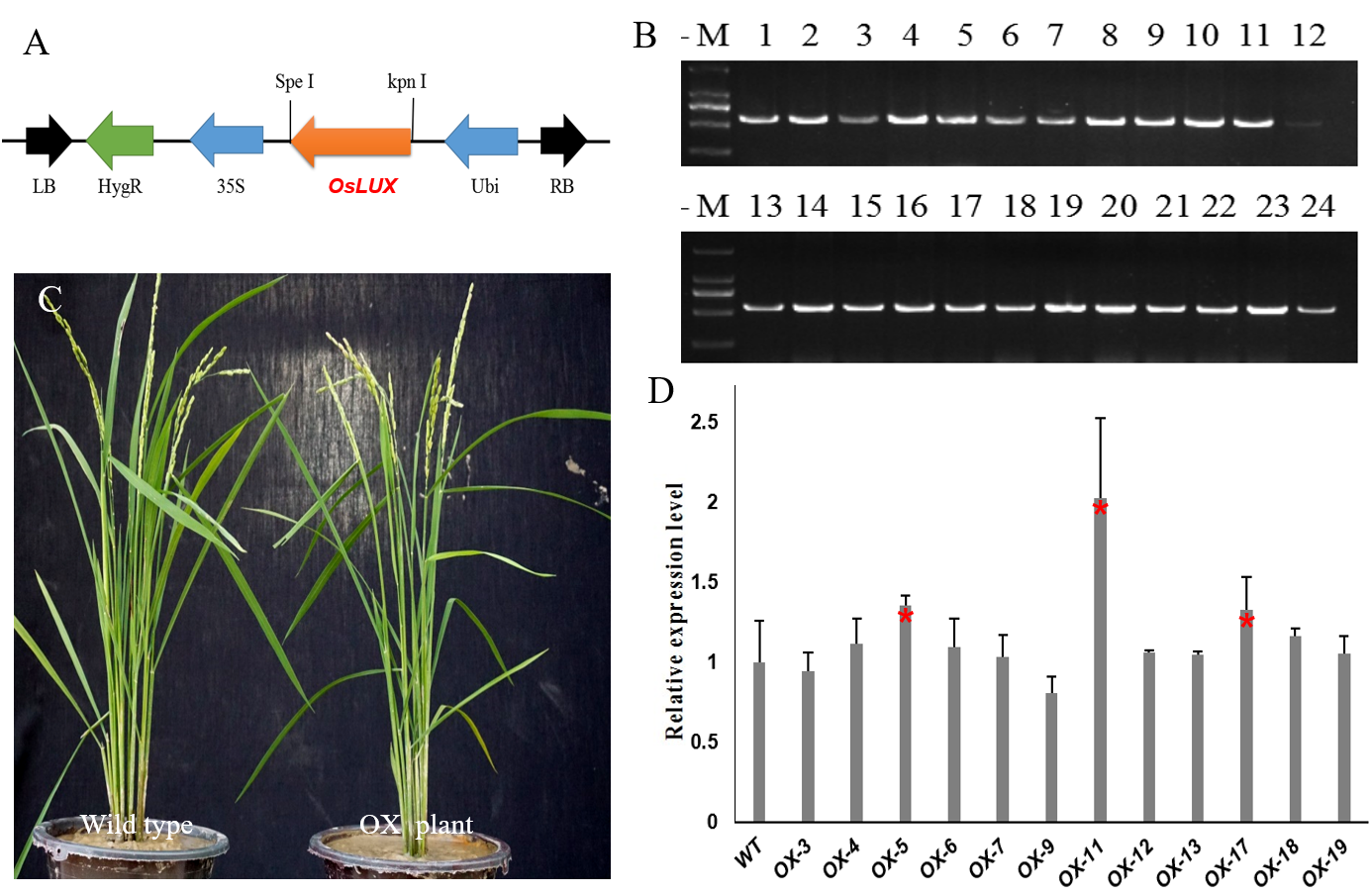


**Figure S4 Constitutive overexpression of *OsLUX*.**

(a) Schematic diagram of *OsLUX* overexpression vector; (b) PCR detection of transgenic lines (Lane M：DL2000); (c) wild-type and *OsLUX* overexpression (OX) plants. (d) qRT-PCR analysis of *OsLUX* expression levels in the leaf of *35S*:*OsLUX* lines (OX); The difference between WT wild-type control and OX were statistically significant (*P* < 0.05, Student’s *t*-test).


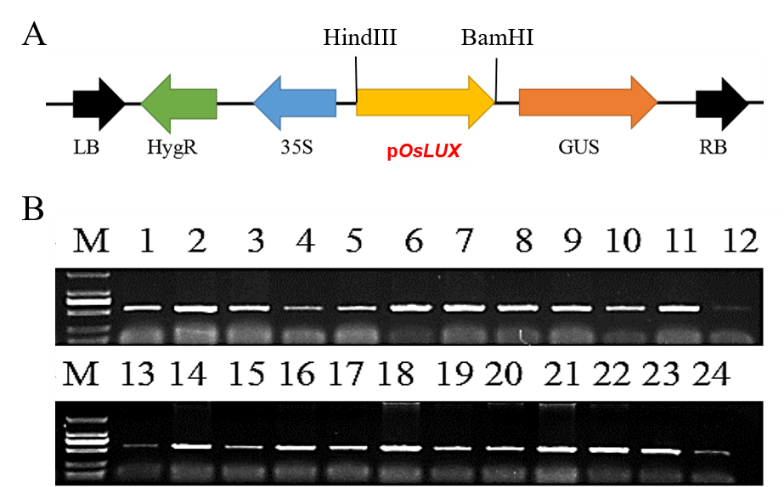


**Figure S5 GUS analysis of *OsLUX* expression pattern.**

(a) Schematic diagram of vector for GUS analysis of *OsLUX* expression; (b) PCR detection of transgenic lines (Lane M：DL2000).


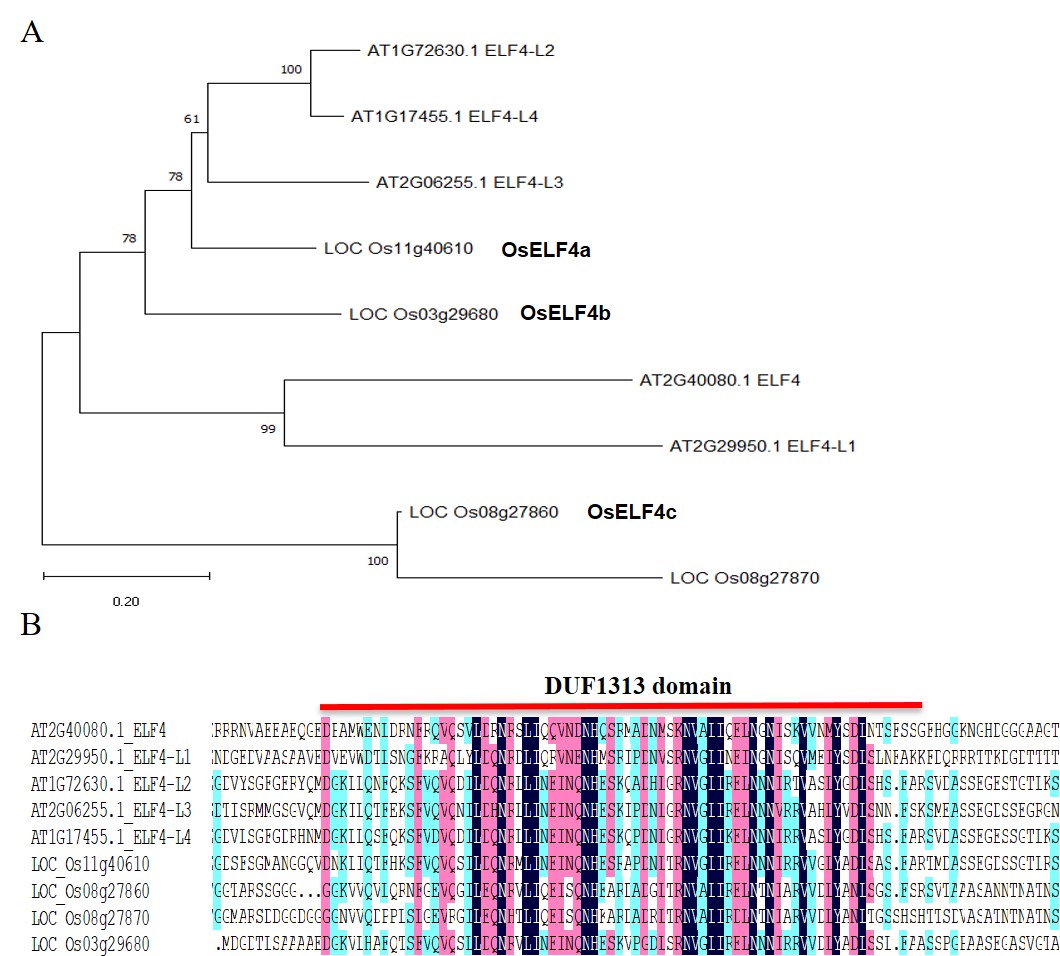


**Figure S6** **Evolutionary relationships of plant ELF4 and ELF4-like proteins.**

(a) Phylogenetic tree of selected ELF4 and ELF4-like proteins from rice and *Arabidopsis*. (b) Protein sequence alignment of the DUF1313 domain. The identities of amino acids are indicated by different colors.


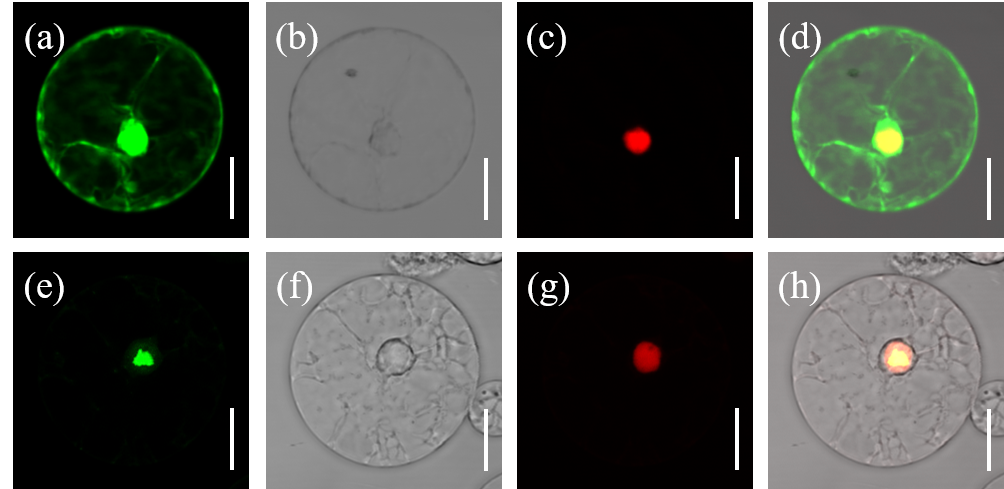


**Figure S7 Subcellular localization of OsLUX.**

(a-d) Ubi-GFP as the control; (e-h) Nuclear localization of OsLUX; (a, e) GFP filter; (b, f) bright field; (c, g) NLS-RFP of nuclear localization control. (d, h) merged images. Scale bars = 100 μm.

Table S1 Primers used for vector construction.

| Primer name | Primer sequence (5′→3′) | |
| --- | --- | --- |
|  | Forward | Reverse |
| Com-OsLUX | cgggatccatttctatgcggtacagtttt | cccaagcttgtatttattcgctattcagttca |
| Cas9-*OsLUX* | caggcggggaggacgagcgggagt | aactcacccgctcgtcctccccgc |
| Cas9-*OsELF4a* | caaccagaaccacgagtcccggg | tgagcatccggttctggtccagg |
| GFP-*OsLUX* | ggggacaagtttgtacaaaaaagcaggcttcatgggcgaggaggcgccgga | ggggaccactttgtacaagaaagctgggtcgtggttggcgtggtggtagt |
| OE-*OsLUX* | atggtaccatgggcgaggaggcgcc | gactagtgtggttggcgtggtggtagt |
| Gus-OsLUX | cccaagcttagctcatctgaacacactgaaga | cgggatccaatactcggattttcttctca |
| NE-/CE-OsLUX | ggggacaagtttgtacaaaaaagcaggcttcatgggcgaggaggcgccgga | ggggaccactttgtacaagaaagctgggtcgtggttggcgtggtggtagt |
| NE-OsELF3a | ggggacaagtttgtacaaaaaagcaggcttcatggcgacgaggggaggag | ggggaccactttgtacaagaaagctgggtcatcatctcgttgccgttcca |
| NE-OsELF3b | ggggacaagtttgtacaaaaaagcaggcttcatgaggggaggaggaggagg | ggggaccactttgtacaagaaagctgggtccgagtcatcttgttgccttt |
| CE-OsELF4a | ggggacaagtttgtacaaaaaagcaggcttcatggaaggtgatagcttct | ggggaccactttgtacaagaaagctgggtcgccgggccggacacgcttc |
| CE-OsELF4b | ggggacaagtttgtacaaaaaagcaggcttcatggacggcgacacgctgtc | ggggaccactttgtacaagaaagctgggtcgtcgaggtgggtggacctga |
| CE-OsELF4c  BD-LUX  BD-ELF4a  AD-ELF3a  AD-ELF3b | ggggacaagtttgtacaaaaaagcaggcttcatggaggaggacagcgtcat  ggaattccatatgatgggcgaggaggcgccgga  ggaattccatatgatggaaggtgatagcttct  gtaccagattacgctcatatgatggcgacgaggggagga  gtaccagattacgctcatatgatgaggggaggaggaggagg | ggggaccactttgtacaagaaagctgggtcctcggtgtcggtggcgcggg  cggaattctcagtggttggcgtggtggt  cggaattcaagcttctagccgggccggacacgct  attcatctgcagctcgagctctcaatcatctcgttgccgttc  attcatctgcagctcgagctcctacgagtcatcttgttgcctttc |
| pRTVcGFP-LUX | tatccagatccagtgggatccatgggcgaggaggcgccgga | agcggccgcactagtaagcttgtggttggcgtggtggtagt |
| pRTVcGFP-ELF4a | tatccagatccagtgggatccatggaaggtgatagcttct | agcggccgcactagtaagcttgccgggccggacacgcttc |
| 1300-myc-ELF3a | caaatcgactctagaaagcttatggcgacgaggggaggag | gagcttttgctccatggtaccatcatctcgttgccgttcca |
| 1300-myc-ELF3b | caaatcgactctagaaagcttatgaggggaggaggaggagg | gagcttttgctccatggtacccgagtcatcttgttgccttt |

Notes: COM-OsLUX, complementation test of *OsLUX*; OE-LUX, over-expression of *OsLUX*; GUS-LUX, promoter activity of *OsLUX*; Cas9-LUX and Cas9*-*OsELF4a, function analysis of *OsLUX* and *OsELF4a* by CRISPR/Cas9 edition; GFP-LUX, subcellular localization analysis of OsLUX; NE-/CE-OsLUX, NE-OsELF3a, NE-OsELF3b, CE-OsELF4a, CE-OsELF4b and CE-OsELF4c, bimolecular fluorescence complementation assay; BD-LUX, BD-ELF4a, AD-ELF3a and AD-ELF3b, Yeast Two-Hybrid assay; pRTVcGFP-LUX, pRTVcGFP-ELF4a, 1300-myc-ELF3a and 1300-myc-ELF3b, Co-IP assay.

Table S2 Primers used for quantitative RT-PCR analysis.

| Gene | Primer sequence (5′→3′) | |
| --- | --- | --- |
|  | Forward | Reverse |
| *UBQ* | AACCAGCTGAGGCCCAAGA | ACGATTGATTTAACCAGTCCATGA |
| *OsLUX* | ACTACTCCATAACGAAGGTGTTTC | ACAAACAAACAGCGTGGATGG |
| *OsELF3a* | TGTCGCCCCTTCGTCAA | GGTCTTTTCCCCAGCTCATT |
| *OsELF3b* | GCTGGATGGCATTTGACTG | ATTTGCGACTTTGAGGGACTA |
| *OsPRR37* | GGGAAGGACTTGGAAATAG | AGCAGCTCGAACACTTGACT |
| *OsPRR95* | CGCTCAGTGGCAGTGTCTGT | GGTATCGCACCTTCTTCTCA |
| *OsGI* | TGGAGAAAGGTTGTGGATGC | GATAGACGGCACTTCAGCAGAT |
| *OsLHY* | CAGATAAGGCCGACACCAAAC | GGTGTGTTGGAACCACATG |
| *Ghd7* | ATATTGTGGGAGCACGTT | ATCTGAACCATTGTCCAAGC |
| *Ehd1* | GCGCTTTTGATTTCCTGC | TTCGGAATATGTGCTGCC |
| *Hd3α* | GCTCACTATCATCATCCAGCATG | CCTTGCTCAGCTATTTAATTGCATAA |
| *RFT* | TACTTCAACTGCCAGCGCGAGG | AGCTATAGCTGCTGCATGCATGGA |

Table S3 Inheritance mode of *lvp2-1* in three cropping seasons in Fuzhou.

| Year and cropping season | Population | Normal plants | *lvp2-1* | X^2^（3:1） |
| --- | --- | --- | --- | --- |
| 2007 (Late) | 300 | 216 | 84 | 1.28 |
| 2008 (Middle) | 249 | 178 | 71 | 1.64 |
| 2008 (Early) | 210 | 159 | 51 | 0.32 |

(*X^2^_0.05，1_*=3.84)

Table S4 Main agronomic traits of a genetic complementation plant.

| Traits | Complementary line |
| --- | --- |
| Heading date (NSD, d) | 64.9±0.7 |
| Heading date (SD, d) | 62.2±1.9 |
| Heading date (NLD, d) | 88.9±2.1 |
| Heading date (LD, d) | 90.8±1.4 |
| Length of flag leaf (cm) | 33.07±3.89 |
| Width of flag leaf (cm) | 1.41 |
| Leaf number | 8-9 |
| Culm length (cm) | 51.3±5.87 |
| Tiller/panicle number | 7.2±0.7 |
| Panicle length (cm) | 14.15±2.35 |
| Grain no. per panicle | 105.51 |
| Kilo-grain weight (g) | 20.81 |

Note: Means ± SD (n > 20).

Table S5 Results of *OsLUX* editing.

| Phenotype | lines | DNA sequence | ORF | Protein |
| --- | --- | --- | --- | --- |
| WT | CK | cggggaggacgagcgggtgatg | 717 base | 238aa |
| *lvp2-2* | 1, 2 | cggggaggacgagcggg***g***tgatg | Ins 56^th^ | 18aa+new peptide |
| *lvp2-3* | 3 | cggggaggacgagcggg***a***tgatg | Ins 56^th^ | 18aa+new peptide |
| *lvp2-4* | 4-6 | cggggaggacgagcggg***t***tgatg | Ins 56^th^ | 18aa+new peptide |
| *lvp2-5* | 7-9 | cggggaggacgagcgg-tgatg | Del 55^th^, PTT | 18aa |
| *lvp2-6* | 10 | cgg-------(16-base)-----atg | Del 42^nd^-57^th^, PTT | 14aa+new 12aa |
| *lvp2-7* | 11 | gagtacg-(274-base)-ggagg | Del 30^th^-303^th^, PTT | 10aa+new 38aa |

aa, amino acid; PTT, premature transcription termination.

Table S6 Results of *OsELF4a* editing.

| Phenotype | lines | DNA sequence | ORF | Protein |
| --- | --- | --- | --- | --- |
| WT | CK | accagaaccggatgctcatcaacga  gatcaaccagaaccacgagtcccgg | 354 base | 118aa |
| *Oself4a-1* | 4/13/21/22 | acc-------(44-base)-------cgg | Del 107^th^-150^th^ | 35aa+new peptide |
| *Oself4a-2* | 6 | acc-------(43-base)------ccgg | Del 107^th^-149^th^ | 35aa+new peptide |
